# The Role of lncRNAs TAPIR-1 and -2 as Diagnostic Markers and Potential Therapeutic Targets in Prostate Cancer

**DOI:** 10.3390/cancers12051122

**Published:** 2020-04-30

**Authors:** Maik Friedrich, Karolin Wiedemann, Kristin Reiche, Sven-Holger Puppel, Gabriele Pfeifer, Ivonne Zipfel, Stefanie Binder, Ulrike Köhl, Gerd A. Müller, Kurt Engeland, Achim Aigner, Susanne Füssel, Michael Fröhner, Claudia Peitzsch, Anna Dubrovska, Michael Rade, Sabina Christ, Stephan Schreiber, Jörg Hackermüller, Jörg Lehmann, Marieta I. Toma, Michael H. Muders, Ulrich Sommer, Gustavo B. Baretton, Manfred Wirth, Friedemann Horn

**Affiliations:** 1 Institute of Clinical Immunology, Medical Faculty, University of Leipzig, Johannisallee 30, D-04103 Leipzig, Germany; Karolin.wiedemann@izi.fraunhofer.de (K.W.); Kristin.Reiche@izi.fraunhofer.de (K.R.); Gabriele.Pfeifer@medizin.uni-leipzig.de (G.P.); Ivonne.Zipfel@izi.fraunhofer.de (I.Z.); Stefanie.Binder@izi.fraunhofer.de (S.B.); ulrike.koehl@izi.fraunhofer.de (U.K.); Friedemann.Horn@izi.fraunhofer.de (F.H.); 2 Department of Diagnostics, Fraunhofer Institute for Cell Therapy and Immunology, RIBOLUTION Biomarker Center Perlickstr. 1, D-04103 Leipzig, Germany; shpuppel@googlemail.com (S.-H.P.); Michael.Rade@izi.fraunhofer.de (M.R.); Sabina.Christ@izi.fraunhofer.de (S.C.); 3 Molecular Oncology, Medical School University of Leipzig, Semmelweisstr. 14, D-04103 Leipzig, Germany; Gerd.Mueller@medizin.uni-leipzig.de (G.A.M.); Kurt.Engeland@medizin.uni-leipzig.de (K.E.); 4 Department of Chemistry and Biochemistry, University of California at Santa Cruz, 1156 High Street, Santa Cruz, CA 95064, USA; 5 Clinical Pharmacology, Rudolf-Boehm-Institute for Pharmacology and Toxicology, Faculty of Medicine, Leipzig University, Härtelstr. 16–18, D-04107 Leipzig, Germany; achim.aigner@medizin.uni-leipzig.de; 6 Department of Urology, University Hospital and Faculty of Medicine, Technische Universität Dresden, Fetscherstr. 74, D-01307 Dresden, Germany; Susanne.Fuessel@uniklinikum-dresden.de (S.F.); michael.froehner@ediacon.de (M.F.); Manfred.Wirth@uniklinikum-dresden.de (M.W.); 7 Zeisigwaldklinik BETHANIEN, Zeisigwaldstraße 101, D-09130 Chemnitz, Germany; 8 National Center for Tumor Diseases (NCT), Partner Site Dresden, German Cancer Research Center (DKFZ), D-69120 Heidelberg, Germany; claudia.peitzsch@nct-dresden.de; 9 OncoRay—National Center for Radiation Research in Oncology, Faculty of Medicine and University Hospital Carl Gustav Carus, Technische Universität Dresden, Helmholtz-Zentrum Dresden—Rossendorf, D-01307 Dresden, Germany; anna.dubrovska@oncoray.de; 10 German Cancer Consortium (DKTK), Partner Site Dresden, German Cancer Research Center (DKFZ), D-69120 Heidelberg, Germany; 11 Helmholtz-Zentrum Dresden—Rossendorf, Institute of Radiooncology—OncoRay, D-01328 Dresden, Germany; 12 Helmholtz Centre for Environmental Research—UFZ, Young Investigators Group Bioinformatics & Transcriptomics, Permoserstr. 15, D-04318 Leipzig, Germany; stephan.schreiber@ufz.de (S.S.); joerg.hackermueller@ufz.de (J.H.); 13 Department of Therapy Validation, Fraunhofer Institute for Cell Therapy and Immunology, GLP Test Facility, Perlickstr. 1, D-04103 Leipzig, Germany; joerg.lehmann@izi.fraunhofer.de; 14 Institute of Pathology, University Hospital and Faculty of Medicine, Technische Universität Dresden, Fetscherstraße 74, D-01307 Dresden, Germany; marieta.toma@ukbonn.de (M.I.T.); michael.muders@ukbonn.de (M.H.M.); Ulrich.Sommer2@Uniklinikum-Dresden.de (U.S.); Gustavo.Baretton@uniklinikum-dresden.de (G.B.B.); 15 Institute of Pathology, Universitätsklinikum Bonn, Venusberg-Campus 1, D-53127 Bonn, Germany; 16 Rudolf-Becker-Laboratory for Prostate Cancer Research, Institute of Pathology, Universitätsklinikum Bonn, Venusberg-Campus 1, D-53127 Bonn, Germany

**Keywords:** lncRNA, prostate cancer, diagnostic marker, therapeutic target, p53, cell cycle arrest, radiation resistance

## Abstract

In search of new biomarkers suitable for the diagnosis and treatment of prostate cancer, genome-wide transcriptome sequencing was carried out with tissue specimens from 40 prostate cancer (PCa) and 8 benign prostate hyperplasia patients. We identified two intergenic long non-coding transcripts, located in close genomic proximity, which are highly expressed in PCa. Microarray studies on a larger cohort comprising 155 patients showed a profound diagnostic potential of these transcripts (AUC~0.94), which we designated as tumor associated prostate cancer increased lncRNA (*TAPIR-1* and *-2*). To test their therapeutic potential, knockdown experiments with siRNA were carried out. The knockdown caused an increase in the p53/TP53 tumor suppressor protein level followed by downregulation of a large number of cell cycle- and *DNA*-damage repair key regulators. Furthermore, in radiation therapy resistant tumor cells, the knockdown leads to a renewed sensitization of these cells to radiation treatment. Accordingly, in a preclinical PCa xenograft model in mice, the systemic application of nanoparticles loaded with siRNA targeting *TAPIR-1* significantly reduced tumor growth. These findings point to a crucial role of *TAPIR-1* and *-2* in PCa.

## 1. Introduction

Prostate cancer (PCa) is the most commonly diagnosed cancer in men world-wide. The present standard protein-based prostate specific antigen (PSA) screening test is being disputed due to a high rate of false positive findings [1,2,3]. Better marker candidates need to be found. The management of PCa comprises potentially curative treatment using radical prostatectomy as well as radiation and hormone deprivation therapy [4]. Depending on the tumor stage, over 55% of the PCa can be permanently controlled using radiation therapy [5,6,7]. Nevertheless, some patients with high-risk PCa develop local relapse, and for metastatic PCa, curative therapies are not available. To treat those patients, novel therapeutic targets are urgently needed.

Advances in sequencing and functional genomics have shown that large, previously unrecognized segments of the human genome are being transcribed. ENCODE predictions suggest that ~80% of the genome’s DNA has been transcribed into RNA [8]. Yet, only 2–3% of the genome is protein-coding. The vast majority of transcripts are non-coding RNAs (ncRNA) [9,10,11]. According to their size, ncRNAs are classified as small (sncRNA < 200 nt) and long (lncRNAs) transcripts [12]. In contrast to sncRNA, only a few lncRNAs have been shown to be involved in fundamental regulatory processes [13]. Owing to their aberrant expression pattern in many cancer cells, lncRNAs emerge as excellent tumor biomarkers [14,15,16]. Dysregulation of lncRNAs in cancer cells hallmarks major cancer properties [16] like evading growth suppression, sustaining proliferative signalling, inducing angiogenesis, activating invasion and metastasis, resisting cell death and enabling replicative immortality [17]. New tools for cancer therapy are provided by small interfering RNAs (siRNAs), a common form of RNAi-based therapeutics, that silence specific genes associated with cancer progression [18]. RNAi employs small, double-stranded RNA molecules that associate with multiple protein factors to form RNA-induced silencing complex (RISC). RISC in turn, mediates either suppression of translation or degradation of target mRNAs and lncRNAs [19,20,21]. While issues regarding siRNA delivery are still a major bottleneck for the therapeutic use of RNAi, novel nanoparticle-based delivery systems give hope for effective prospects of in vivo applications. SiRNAs, targeting specific cancer-associated RNAs, may be a new class of drugs for cancer treatment, in particular in combination with traditional anticancer treatments to produce synergistic effects [18,22].

In this study, we discovered, via transcriptome-wide next generation sequencing (NGS), a genomic locus encoding two yet uncharacterized lncRNA transcripts ENST00000438247.1 and ENST00000366424.2 (designated here as *TAPIR-1* and *-2*, respectively), which are strongly upregulated in prostate tissue specimens of patients suffering from PCa. The finding was verified using microarray studies on a larger validation cohort comprising 155 patients thus indicating the diagnostic potential of these transcripts. Furthermore, the value of *TAPIRs* to serve as promising therapeutic targets was demonstrated by the following findings: the knockdown of *TAPIRs*: (a) leads to a prominent cell cycle arrest, (b) reduces tumor growth in a xenograft mouse model and (c) results in a renewed sensitization to X-ray treatment.

## 2. Results

### 2.1. TAPIR lncRNAs as a Potent Diagnostic Prostate Cancer Biomarker

**Figure 1:**
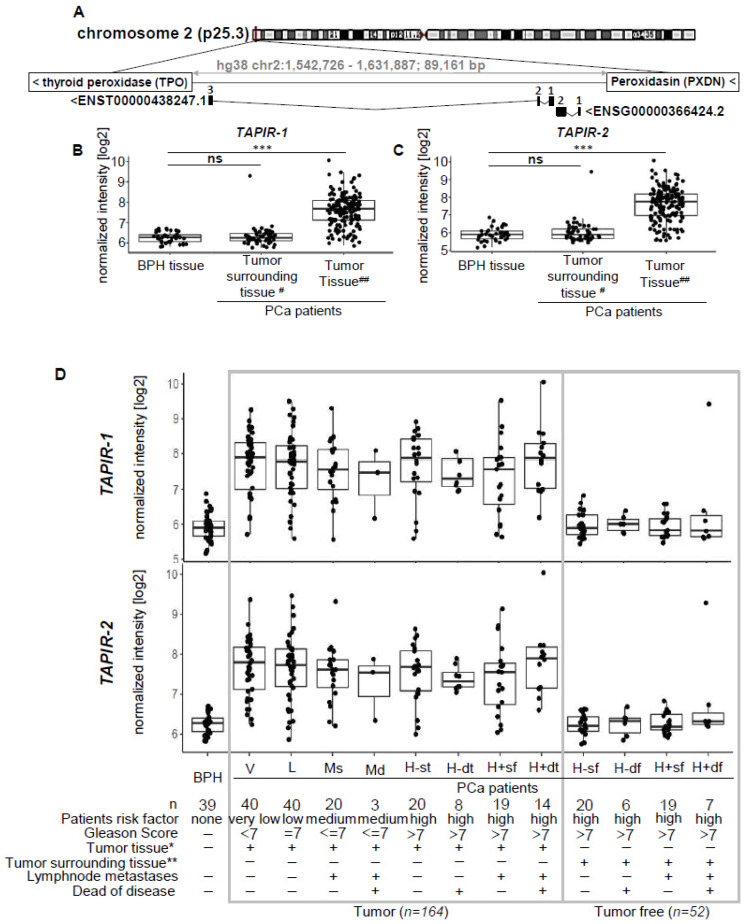
Expression analysis of the lncRNAs *TAPIR-1* and *-2* shows significant overexpression in prostate cancer tissue. (**A**) Schematic representation of the chromosomal location of the *TAPIR-1* and *TAPIR-2* gene locus and intron exon transcript structure. Exons are represented by numbered black boxes, introns by black lines. (**B**,**C**) Box plot analysis for the lncRNAs *TAPIR-1* (**B**), *TAPIR-2* (**C**), measured by Agilent custom expression microarrays of the validation cohort only (tumor tissues from 124 PCa patients and control tissues from 39 BPH patients). The results of the exploratory cohort are shown in Figure S1. (**D**) Expression patterns of *TAPIR-1* (**D**), and *TAPIR-2* (**B**) determined using microarray analyses are shown related to clinical risk classification. Normalized expression intensity [log2] was plotted against subgroups based on clinical data sets: patient risk factor (none, very low, low, and high); Gleason Score (none, =7, ≤7, >7); tumor tissue (−/+), verified tumor cell content >60% for tumor tissue (denoted with *; −/+); matched tumor adjacent tissue (−/+), verified tumor cell content 0–5% for matched tumor surrounding tissue (denoted with **; −/+); lymph node metastases (−/+), died of disease (−/+). Groups are defined as follows: BPH, PCa-risk groups: V = very low; L = low; Ms = medium, with lymph node metastases; Md = medium, with lymph node metastases and death because of disease (DoD); tumor tissue (t): H-st = high, without metastases; H-dt = high, without metastases and DoD; H+st = high with lymph node metastases; and H+dt = high, with metastases and DoD; matched tumor (free) adjacent tissue (f): H-sf = high, without metastases; H-df = high, without metastases and DoD; H+sf = high with lymph node. ***: FDR (false discovery rate) ≤0.001; #: tumor cell content 0–5%; ##: tumor cell content >60%.

**Figure 2:**
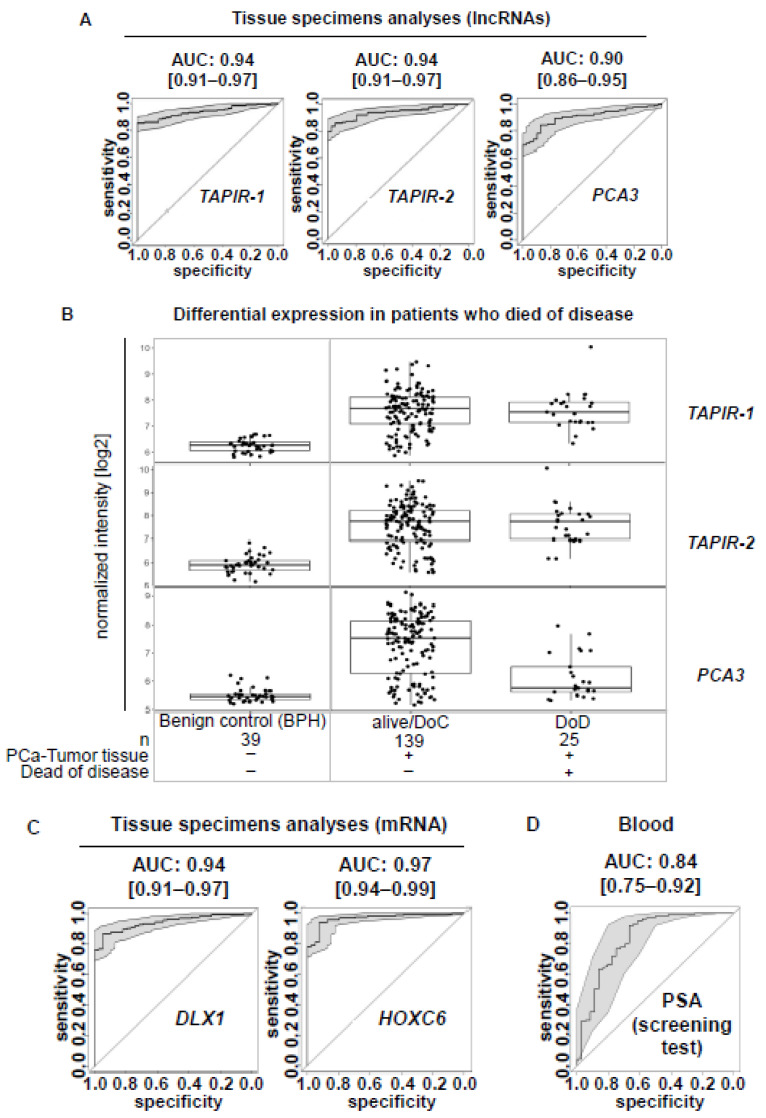
Expression pattern of *TAPIR-1* and *-2* showing potent diagnostic properties as prostate cancer biomarker in tissue analysis. (**A**) ROC curve analysis for the lncRNAs *TAPIR-1*, *TAPIR-2* and the clinical PCa biomarker prostate cancer antigen 3 (PCA3) measured using Agilent custom expression microarray analysis of tissue specimens of the validation cohort (tumor tissues from 124 PCa patients and control tissues from 39 BPH patients). All three RNA markers, *TAPIR-1*, *TAPIR-2*, and *PCA3*, revealed high PCa diagnostic AUC values of 0.94 [CI:0.91–0.97] measured by two specific custom probes; FDR (false discovery rate) ≤ 0.001, of 0.94 [CI:0.91–0.97] measured by three specific custom probes; FDR ≤ 0.001, and of 0.9 [CI:0.86–0.95] measured by three specific custom probes; FDR ≤ 0.01, respectively. (**B**) The validation cohort was stratified into patients who died of the tumor (DoD, *n* = 25) and patients who survived or died of other causes (alive/DoC, *n* = 139). Patients with benign prostate hyperplasia (BPH, *n* = 39) served as control group. Expression patterns of *TAPIR-1*, *TAPIR-2*, and *PCA3*, determined using microarray analyses, are shown related to clinical classification. Normalized expression intensity [log2] was plotted against subgroups. TAPIR-lncRNAs show high expression and a high diagnostic potential even for the DoD patient group (AUC 0.98 [CI:0.96–1] and AUC 0.98 [CI:0.95–1] for *TAPIR-1* and *-2*, respectively) in contrast to PCA3, where the expression was significantly lowered in DoD (AUC 0.80 [CI:0.68–0.91]). (**C**) ROC curve analysis for the mRNA PCa biomarker *DLX1* and HOXC6 (SelectMDx) measured by Agilent custom expression microarray analysis of tissue specimens of the validation cohort (tumor tissues from 124 PCa patients and control tissues from 39 BPH patients). *DLX1* and HOXC6 revealed high PCa diagnostic AUC values of 0.94 [CI:0.91–0.97] and 0.97 [CI:0.94–0.99], respectively. These results indicate that the AUCs of lncRNA *TAPIR-1* and *-2* are in the same range as those mRNA PCa markers and have the potential to serve as highly sensitive and specific diagnostic markers. (**D**) ROC curve analysis of the prostate specific antigen (clinical PSA blood test) revealed an AUC value of 0.837 [CI:0.75–0.92]; FDR ≤ 0.01 in our validation cohort. (**D**) ROC curve analysis of the prostate specific antigen (clinical PSA blood test) revealed an AUC value of 0.837 [CI:0.75–0.92]; FDR ≤ 0.01 in our validation cohort.

To detect novel PCa biomarkers, an exploration cohort comprising tissue specimens from 40 PCa patients, including 16 tumor-adjacent tissues, as well as another 8 benign prostate hyperplasia (BPH) patients were screened using transcriptome-wide deep sequencing (NGS). In total, 2040 differentially expressed genes were found (false discovery rate (FDR)/p.adj < 0.01), 1270 of them being upregulated and 770 being downregulated. Among them we identified an intergenic lncRNA locus on chromosome 2 (p25.3; hg38 chr2:1,546,665-1,625,419; 78,753 bp) encoding two transcripts (ENST00000438247.1 and ENST00000366424.2) (Figure 1A). Here, we refer to them as tumor associated prostate cancer increased lncRNA (*TAPIR-1* and *-2*). They are strongly upregulated in PCa compared to tumor-free and BPH tissue (Figure 1B,C, Figure S1). Both transcripts are spliced (*TAPIR-1*: 3 exons, 786 bp length; *TAPIR-2*: 2 exons, 1,641 bp length) and encoded on the minus DNA strand. The locus is flanked by *thyroid peroxidase* (*TPO*) and *peroxidasin* (*PXDN*) genes. To determine whether *TAPIR-1* and *-2* represent non-coding transcripts, we used CPAT, a tool that predicts the coding potential of the transcript [23]. Based on open reading frame (ORF) size, ORF coverage, Fickett TESTCODE statistic, and hexamer usage bias, a coding probability below cutoff 0.364 denotes non-coding transcripts. Our analysis revealed a coding probability of 0.003 and 0.07 for *TAPIR-1* and *TAPIR-2*, respectively, assigning TAPIRs as ncRNAs (Table S1). Microarray studies on a larger validation cohort comprising 191 samples (124 PCa patients, including 36 tumor-adjacent tissues, and 31 BPH patients) confirmed a high diagnostic potential of both transcripts. (Figure 1B,C) Patient stratification was carried out according to risk factor classes (relying on the clinical parameters: Gleason score, lymph node metastases, and death from the disease). Overexpression of *TAPIR* was detected in all stages, from very low (Gleason score <7) up to high risk patients (Gleason score >7) (Figure 1D). The overall accuracy as a diagnostic PCa biomarker was determined by the area under the receiver operating characteristic (ROC) curve analysis yielding an AUC of 0.94 [CI:0.91–0.97] and 0.94 [CI:0.91–0.97] for *TAPIR-1* and *TAPIR-2*, respectively (Figure 2A). A more specific clinical PCa biomarker that can be determined in urine and tissue specimens is the lncRNA prostate cancer antigen 3 (*PCA3*). In comparison, *PCA3* (measured in tissue) in our cohort revealed an AUC of 0.904 [0.86–0.95] (Figure 2A and Figure S2). Although this value is somewhat lower compared to *TAPIR-1* and *-2*, it still represents a good diagnostic marker. However, significant differences become apparent when our PCa cohort is stratified into patients who died of the tumor (DoD) and patients who survived or died of other causes (alive/DoC) (Figure 2B). Notably, the expression of *PCA3* in PCa tissue was significantly lower in patients who had died of their tumor (AUC of 0.98 [0.96–1] and 0.98 [0.95–1] for *TAPIR-1* and *-2*, respectively versus AUC of 0.80 [0.68–0.91] for *PCA3*). Analyzing the differential mRNA expression obtained from tissue specimens of the diagnostic mRNA markers DLX1 and HOXC6 (derived from the Select MDx urine test, which is used in combination with clinical data) an AUC of 0.94 [0.91–0.97] and 0.97 [0.94–0.99] was revealed, respectively (Figure 2C). The AUC value from the standard PCa screening marker, prostate specific antigen (PSA; protein biomarker; clinical blood test), is only 0.84 [0.75–0.92] (Figure 2D). These results indicate that the AUCs of *TAPIR-1* and *-2* are in the same range and have the potential to serve as highly sensitive and specific diagnostic markers.

### 2.2. TAPIR-1 and -2 Overexpression Is Restricted to Tumor Tissue

Custom expression microarray analysis revealed a strong expression of *TAPIR* in PCa tissue (median log2 intensity ± sd: 7.68 ± 0.77 (*TAPIR-1*), 7.76 ± 0.93 (*TAPIR-2*), 7.70 ± 0.85 (*TAPIR-1* und *-2*); *n* = 164) and low expression in BPH tissue specimens (median log2 intensity ± sd: 6.28 ± 0.25 (*TAPIR-1*), 5.90 ± 0.37 (*TAPIR-2*), 6.09 ± 0.36 (*TAPIR-1* and -2); *n* = 39) and tumor-adjacent tissue (median intensity + sd: 6.26 ± 0.49 (*TAPIR-1*), 5.90 ± 0.58 (*TAPIR-2*), 6.12 ± 0.56 (*TAPIR-1* and *-2*); *n* = 52) (Figure 1B–D). Signal intensity differences between tumor and BPH samples were significant for both *TAPIR-1* and *TAPIR-2* (Benjamini-Hochberg-adjusted *p*-values of matching probe sets less than 9.67E-14 and 1.79E-15, respectively). To visualize *TAPIR*-transcripts ViewRNA^®^ in situ hybridization (ISH) was carried out using formalin-fixed paraffin-embedded prostate tissue sections (exhibiting areas with benign glands, high grade prostate intraepithelial neoplasia (PINs) and PCa tissue at the same section). Specific staining was observed in PINs and PCa areas and no or weak staining in extended normal glands, whereas connective tissues were unstained (Figure S3). The GTEx database, providing RNA-seq data of 53 tissues from 570 healthy donors, was used to determine the expression profile of both *TAPIR* transcripts (Figure S4). *TAPIR-1* and *-2* RNA levels in normal tissues were generally low. Elevated *TAPIR-1* expression was only observed in thyroid samples (*n* = 283), whereas *TAPIR-2* showed broad but low expression in brain cerebellum (*n* = 121), testis (*n* = 165), brain cerebellar hemisphere (*n* = 98), normal breast (*n* = 181) and prostate tissue (*n* = 100). Moreover, the analysis of expression profiles in tumor tissues (11,133 samples from 32 tumor entities) from the Cancer Genome Atlas (TCGA) consortium showed similar expression patterns for both *TAPIR* transcripts (Figure S5). In line with our findings, these data confirmed the strongest expression for both *TAPIR* transcripts in PCa (*n* = 556) and also revealed elevated expression levels in invasive breast cancer (*n* = 1253).

### 2.3. TAPIR Transcript Knockdown Causes Downregulation of Cell Cycle Genes and Growth Arrest in Prostate Tumor Cells

**Figure 3:**
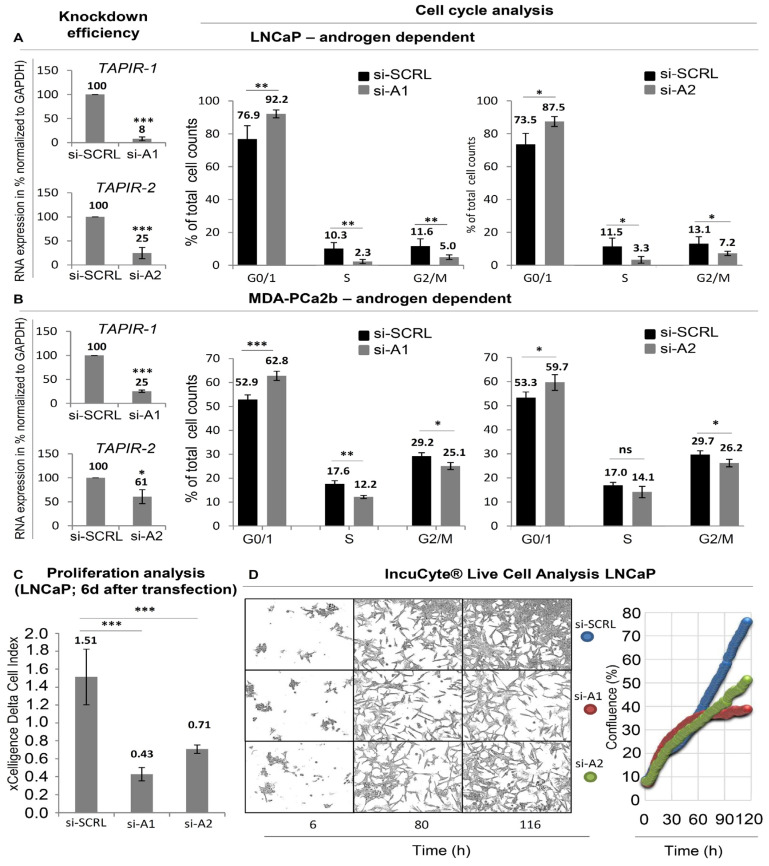
*TAPIR* transcript knockdown causes downregulation of cell cycle genes and growth arrest in PCa cells. (**A**) LNCaP and (**B**) MDA-PCa2b cells were cultured and treated with siRNA to knockdown *TAPIR-1* and *-2*. The knockdown efficacy of siRNAs is shown 72 h after transfection. RNA was isolated from LNCaP and MDA-PCa2b cells and reverse transcribed into cDNA. The expression levels of *TAPIR-1* and *-2* were determined using qPCR and normalized to *GAPDH*. Data represent the mean ± s.d. of *n* = 3 biological replicates. Significance *p* ≤ 0.001 (***); student-*t* test. Cell cycle analysis was carried out after *TAPIR* knockdown. For cell cycle analysis, LNCaP and MDA-PCa2b cells were transfected with siRNAs against *TAPIR-1* (si-A1), *TAPIR-2* (si-A2) and a scrambled control (SCRL), fixed after 72 h and 120 h, DNA content labelled with propidium iodide and measured using flow cytometry. G0/1 phase arrest after *TAPIR* knockdown was observed. Data represent the mean ± s.d. of at least *n* = 4 biological replicates. Significance *p* ≤ 0.05 (*), *p* ≤ 0.01 (**); two-sided student-*t* test. (**C**) xCelligence real-time cell analysis (RTCA) proliferation analysis. The xCELLigence^®^ RTCA (Roche, Basel, Switzerland) instrument was used to non-invasively monitor cell status. SiRNA-transfected LNCaP cells were seeded and cultured in an electronic 96-well microtiter plate. The impedance value was measured and is plotted, which shows that as the cell index increases the cells proliferate. Data represent the mean ± s.d. of *n* = 3 biological replicates performed in technical quadruplicate. Significance *p* ≤ 0.001 (***); student-*t* test. (**D**) IncuCyte live cell analysis. LNCaP cells were siRNA-transfected and seeded into 96-well ImageLock plates. Cells were grown for six days and monitored using time-lapse microscopy hourly (representative images for 12 h, 60 h, and 116 h are shown). The results of the IncuCyte experiments were quantified using cell confluency and are shown by blue, red, and green filled circles indicating SCRL, si-A1, and si-A2, respectively.

To unravel the therapeutic potential of *TAPIR-1* and -2, four different specific siRNAs derived from *TAPIR-1* and *-2* (si-A1, si-B1, si-A2, siB2; Table 1) were used to knockdown the TAPIR-transcripts in LNCaP and MDA-PCa2b cells (Figure 3 and Figure S6). When comparing between primary tumors and cell lines (LNCaP, MDA-PCa2b), the expression of TAPIR lncRNAs in the cell lines is low (Figure S7). However, these cells are still suitable as a model to study the phenotype after *TAPIR* knockdown.

As seen in Figure 3A,B, knocking down *TAPIR-1* and *-2* transcripts induces a growth arrest in PCa cell line LNCaP and MDA-PCa2b. A 70% and 50% inhibition of cell proliferation after *TAPIR-1* and *TAPIR-2* knockdown, respectively, was observed using impedance-based xCELLigence real-time cell analysis (RTCA) assay (Figure 3C). RTCA allows a non-invasive growth monitoring of adherent cells after six days post transfection but does not exclude effects derived from changes in cell shape, size and attachment. Therefore, live cell imaging was performed and quantified using cell confluency (Figure 3D). While the cell shape remained largely unaltered upon *TAPIR-1* and *-2* knockdown, decreased cell densities were observed, especially five days (116 h) after transfection. Cell cycle analysis revealed an enrichment of cells in G0/G1 accompanied by an equivalent decrease in S and G2/M phases by 50% upon si-A1-mediated *TAPIR-1* knockdown. *TAPIR-2* knockdown revealed similar but less profound effects on cell cycle distribution. Similar results were observed for MDA-PCa2b cells. Additional experimental evidence of the specificity of the siRNAs could be obtained by DU145 cells that do not express significant levels of *TAPIR-1* or *-2* lncRNA transcripts. Transfection of siRNAs (siA1 and siA2) in these cells does not alter their proliferation and does not lead to increased levels of p21^CIP1^/*CDKN1A* repression or repression of cell cycle key regulators like *CCNB1* and *CDC25C* (Figure S7). We also knocked down the androgen receptor in LNCaP cells and determined the expression of *TAPIR-1* and *-2* (see Figure S8). As a result, we showed a slight increase in TAPIR expression with loss of androgen sensitivity.

### 2.4. Increase in p53 Levels and Downregulation of Cell Cycle- and DNA-Repair Control Genes Following TAPIR Knockdown

**Figure 4:**
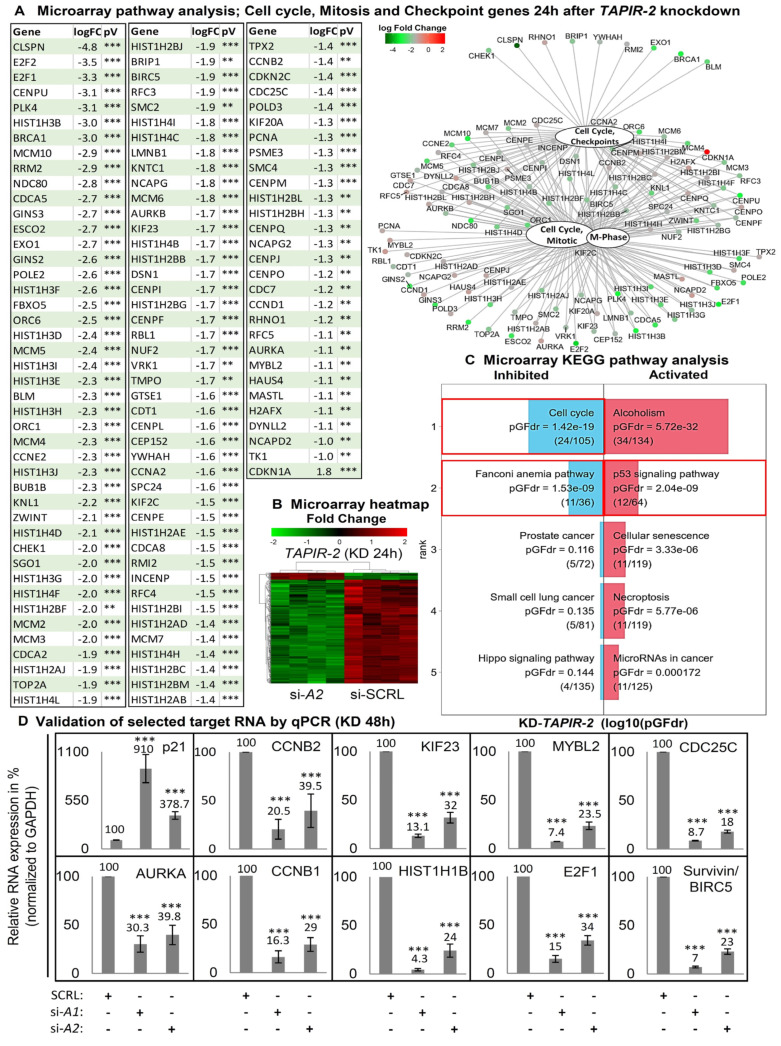
Increase in p53 levels and downregulation of cell cycle and DNA repair control genes following *TAPIR-2* knockdown. (**A**) Differential mRNA expression following *TAPIR-2* knockdown. A microarray analysis was performed 24 h after *TAPIR-2* knockdowns. Data were analyzed and visualized using ReactomePA software to identify regulated target genes and cellular processes. ReactomePA is a pathway database tool used for the visualization, interpretation, and analysis of regulated pathways. A table shows all cell cycle, mitosis, and checkpoint regulated genes 24 h after *TAPIR-2* knockdown. Notably, this table includes 117 of the total 276 significantly regulated genes. Adjusted *p*-values are given as *p* ≤ 0.01 (**) and *p* ≤ 0.001 (***). Regulatory interactions are visualized using the function cnetplot of the ReactomePA package. An enlarged version of the graph is provided as Figure S9. Downregulated genes are shown in green, upregulated genes in red. Shown are the identified regulated genes of the four most regulated cellular processes: cell cycle, mitotic, m-phase, and cell cycle checkpoints. Interactions of molecules are indicated by black lines. (**B**) Heatmap of differentially up- and down-regulated *TAPIR-2* target genes 24 h post siRNA transfection, determined using microarray analysis (performed in quadruplicates). Significant upregulated genes are shown in red, downregulated genes in green. Note that ~95% of the targets are downregulated. (**C**) Microarray signaling pathway impact analysis (SPIA). Transcriptome wide RNA expression patterns of siRNA treated LNCaP cells (each experiment was performed in quadruplicates) were measured using custom expression microarrays 24 h after transfection. The biological pathways extraction was done using the Kyoto Encyclopedia of Genes and Genomes (KEGG) PATHWAY database. Downregulation of cell cycle and Fanconi anemia pathway genes (left red boxes) and upregulation of the p53 signaling pathway (right red box) is shown. The false discovery rate-adjusted global probability (pGFdr) is given. (**D**) Validation of the microarray-determined expression patterns of cell cycle regulating target RNAs after *TAPIR* knockdown using qPCR. Total RNA was isolated from LNCaP cells 48 h post siRNA transfection and reverse transcribed into cDNA. qPCR was performed to quantify p21^CIP1^/CDKN1A, *CCNB1*, *CCNB2*, *KIF23*, *MYBL2*, *CDC25C*, *AURKA*, *HISTH1B*, *E2F1*, and Survivin/*BIRC5*. The ∆Ct values were determined by subtracting the Ct value of the housekeeper Ct value (GAPDH). Each qPCR reaction was performed in triplicate with at least three independent biological replicates. Data are shown as means ± s.d.; Significance *p* ≤ 0.001 (***); two-sided student-*t* test. Similar data are obtained for *TAPIR-1* knockdown, shown in Figures S10 and S11.

**Figure 5:**
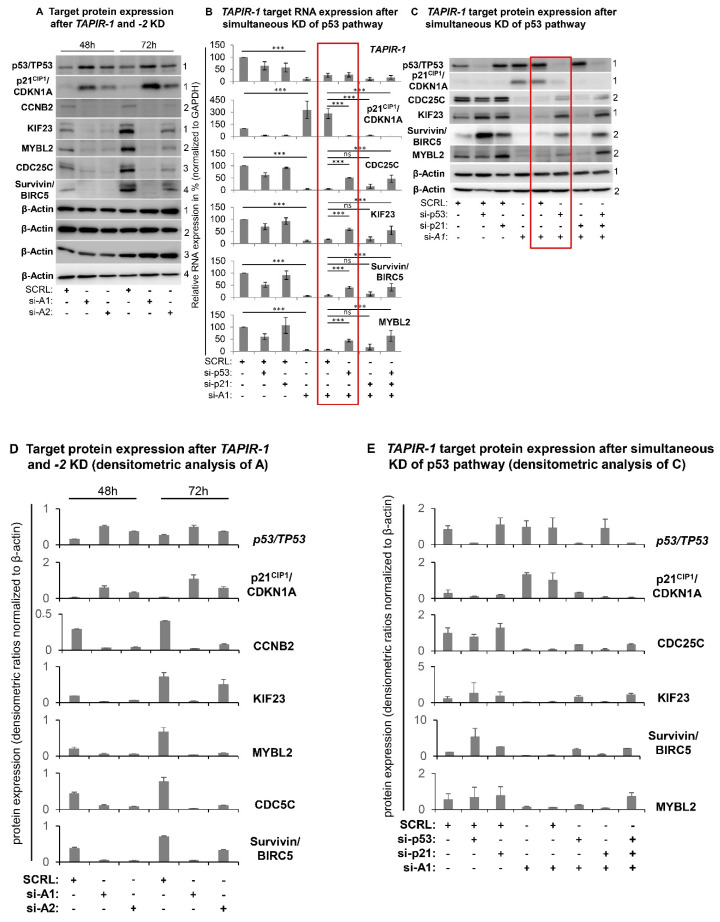
*TAPIR-1* knockdown induces the p53-DREAM pathway. LNCaP cells were transfected with siRNAs against *TAPIR-1*, *TAPIR-2*, p53/*TP53*, p21^CIP1^/*CDKN1A* (p21), and a scrambled control (SCRL) and cultured for 48 h and 72 h. (**A**) Expression pattern of cell cycle regulating target proteins after *TAPIR-1* knockdown. Post siRNA transfection protein lysates were prepared at 48 h and 72 h. SDS-PAGE and western blot were performed to detect upregulation of *p53* and *p21^CIP1^* and downregulation of *CDC25C*, cyclin B2/*CCNB2*, *KIF23*, *MYBL2*, and Survivin/*BIRC-5* protein targets. *β-actin* was used as loading control. One of two independent experiments with equal results is shown. (**B**) Results of qPCR-based expression analysis of different genes 48 h post transfection with siRNAs directed against *TAPIR-1* (si-A1), p53/*TP53*, and p21^CIP1^/*CDKN1A* are shown. The ∆Ct values were determined by subtracting the Ct value of the housekeeper Ct value (GAPDH). Each qPCR reaction was performed in triplicate with at least three independent biological replicates. Data are shown as means ± s.d.; significance *p* ≤ 0.001 (***), ns = non-significant; two-sided student-*t* test. (**C**) Western blot analyses of different proteins 72 h post transfection with siRNAs directed against *TAPIR-1* (si-A1), p53, and p21^CIP1^/*CDKN1A* are shown. β-actin served as loading control. One of two independent experiments with consistent results is shown. Uncropped western blot images are shown in Figure S15. Red box denotes rescue effect of p53 knockdown after *TAPIR-1* knockdown. Densitometry analysis of each band and intensity ratio calculations (target/GAPDH) are shown for western blot analysis of subparagraph A and C in (**D**) and (**E**), respectively.

Microarray analysis 24 h after *TAPIR-1* and *-2* knockdown showed a downregulation of a broad spectrum of cell cycle genes, comprising all central regulators (Figure 4A–D; Figure S10A–C; table of all regulated genes in supporting excel file S1 and S2). Gene Ontology (GO) term enrichment analysis revealed a significant regulation of gene sets responsible for cell cycle progression and mitosis (Tables S2–S4). *TAPIR-1* and *-2* knockdown also led to an upregulation of the p53-signaling- (pGFdr = 0.000217) and Fanconi anemia DNA-repair pathway (pGFdr = 1.1^−6^) (Figure 4C; Figure S10C). Importantly, essentially all genes substantially deregulated are targets of the DREAM transcriptional repressor complex [24,25]. To follow up on the activation of the *p53* pathway, we analyzed *p53* protein stability levels, as its expression is mostly regulated at the protein level. *p53* protein accumulated 48 h and 72 h post *TAPIR-1* and *-2* siRNA transfection (Figure 5A), and the expression of *p21^CIP1^/CDKN1A* was induced at the transcriptional and protein levels (Figure 4D; Figure 5A,D) in LNCaP cells. Furthermore, a large number of key genes controlling the cell cycle were downregulated upon *TAPIR* knockdown, e.g., *AURKA*, *MYBL2*, *CDC25C*, *CCNB1*, *CCNB2*, *E2F1*, *FOXM1*, *HIST1H1B*, *KIF23*, and *MKI67* (Figure 4A,D, Figure 5A). Several of the *TAPIR*-knockdown targets have been shown to be DREAM targets in previously published studies, e.g., *MYBL2*, *CDC25C*, *CCNB1*, and *CCNB2* [26,27,28,29]. Survivin/*BIRC-5*, an inhibitor of apoptosis, which is highly expressed in most human tumors and fetal tissue, but completely absent in terminally differentiated cells, was also found to be suppressed. Importantly, most of the siRNA-dependent knockdown targets have been shown in a genome-wide study to belong to the *p53*-DREAM pathway [25]. The same regulatory pattern was found in MDA-PCa2b cells for all target genes, with the exception of p21^CIP1^/*CDKNA1* (Figure S12).

Moreover, genes that control the cellular DNA repair system are also regulated by the knockdown. We found a downregulation of *BRCA1* (breast cancer 1, early-onset), *CHEK1* (Checkpoint kinase 1), and *CLSPN* (Claspin). Furthermore, numerous key regulators of the DNA-damage response and of the Fanconi anemia pathway were found to be downregulated, suggesting that the DNA repair system is inhibited in response to *TAPIR* knockdown. All these genes are described as validated p53-DREAM repressed target genes [24].

### 2.5. TAPIR Knockdown Activates the p53-DREAM Tumor Suppressor Pathway

To investigate the impact on the p53/TP53 pathway we carried out TAPIR knockdown experiments using the most efficient *TAPIR-1* knockdown (si-A1) in combination with p53 and/or p21CIP1 knockdown. Co-transfection with siRNAs directed against p53/TP53 or p53/p21CIP1/CDKN1A abrogated downregulation of cell cycle genes at the RNA and protein level (Figure 5B,C,E; Figures S13 and S14). Although siRNA-directed knockdown of p21CIP1/CDKN1A was efficient, downregulation of cell cycle genes could not be prevented in LNCaP cells. Furthermore, in MDA-PCa2b cells an upregulation of p21CIP1/CDKNA1 after *TAPIR-1* and *-2* knockdown was not observed 72 h after siRNA transfection (Figure S12). In conclusion, p21CIP1 function seems not to be crucial in TAPIR knockdown-induced cell cycle arrest.

### 2.6. TAPIR Represents a Potential Therapeutic Target in Prostate Cancer

**Figure 6:**
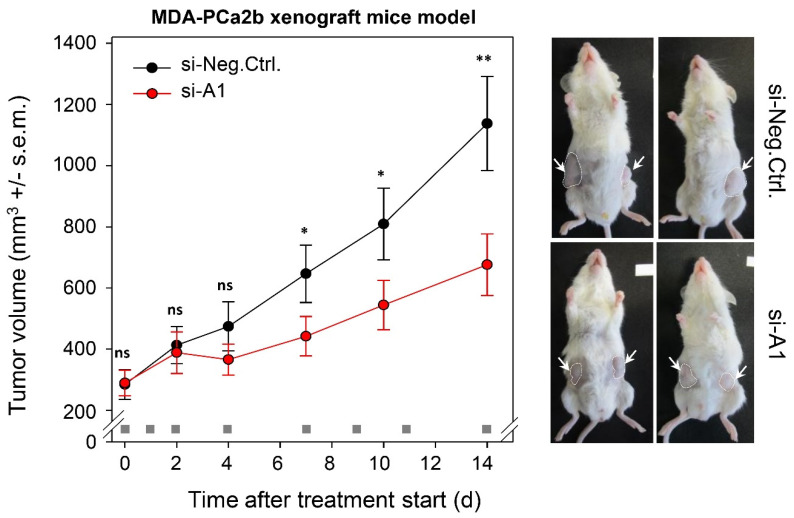
Treatment with nanoparticle/*TAPIR-1* siRNA complexes reduces tumor growth in a PCa xenograft model in vivo. To generate PCa tumor xenografts, MDA-PCa2b cells were injected subcutaneously in 6–8-week-old immunodeficient mice (NOD/SCID/IL2r gamma (null)) after shaving the injection region. When the tumors were established after 5 weeks, mice were randomized into treatment (PEI/siA1) and control groups (PEI/siCtrl) with *n* = 13 tumors per group. Mice were treated at the time points indicated in the figure (gray square) using intraperitoneal injection of PEI F25-LMW/siRNA complexes (complexes containing 10 µg siRNA; ~0.4 mg siRNA/kg body weight). Tumor volumes were measured in all three dimensions (length × width × height). After 14 d mice were sacrificed. The knockdown efficacy in vivo was 30% (RT-qPCR). Significance compared to control (si-Neg.Ctrl.): *p* ≤ 0.05 (*), *p* ≤ 0.01 (**), ns = non-significant; student-*t* test.

By applying a preclinical in vivo tumor xenograft model, we tested the potential of TAPIR as a therapeutic target for PCa. To this end, human MDA-PCa2b cells were subcutaneously injected in mice, and upon establishment of s.c. xenografts with solid growth kinetics, treatment was started. TAPIR-1 siRNA was formulated by complexation with polyethylenimine (PEI). These PEI/siRNA nanoparticles, based on low molecular weight PEI F25-LMW [30] have been found previously to be stable in biological media containing proteins and to allow frozen storage [31]. Moreover, they have been extensively explored in therapeutic knockdown studies of various protein-encoding oncogenes (see, e.g., [32,33,34,35,36] and for therapeutic miRNA replacement [37]). Previous biodistribution studies also demonstrated efficient PEI-mediated siRNA delivery into s.c. tumor xenografts upon intraperitoneal injection [38]. Here, for the first time this mode of administration was also employed to targeting a noncoding RNA. Indeed, the systemic application of *TAPIR-1*-siRNA formulated in the PEI-based nanoparticles led to a significant ~40% reduction of the tumor growth (Figure 6). After completing the experiment, mean tumor sizes in mice treated with *TAPIR-1*-siRNA and scrambled siRNA amounted to 676.38 mm^3^ and 1138.00 mm^3^, respectively (*p* < 0.01).

### 2.7. TAPIR-1 and -2 Knockdowns in Radiation-Resistant Prostate Cancer Cells Confer Renewed Sensitization to X-ray Treatment

**Figure 7:**
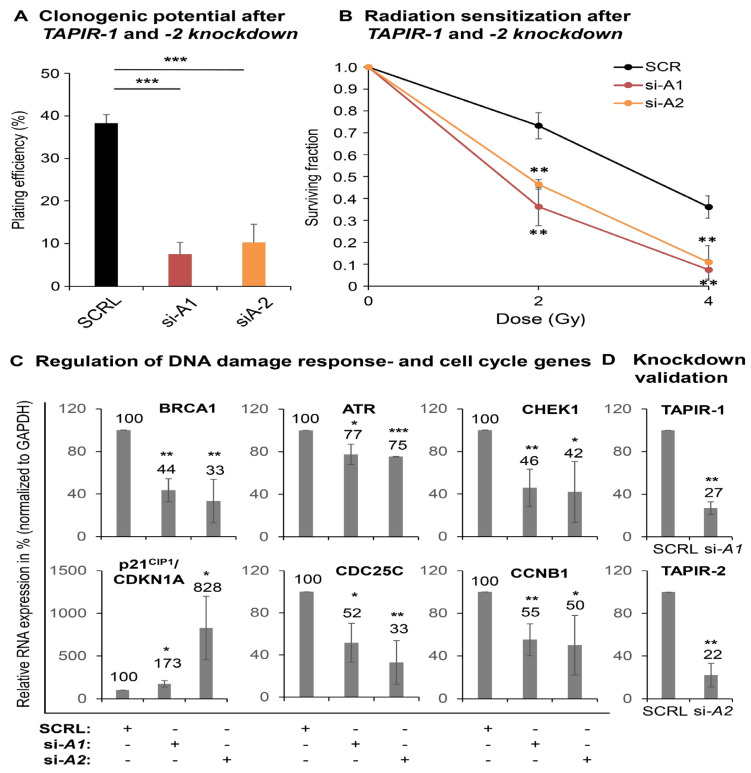
*TAPIR* knockdowns in radiation-resistant prostate cancer cells (LNCaP-RR) results in a renewed sensitization to X-ray treatment. The radioresistant LNCaP cell subline was used to analyze the effect of *TAPIR* knockdown on proliferation (colony formation assay) after X-ray treatment. Cells were treated with siRNA targeting *TAPIR-1* or *TAPIR-2* and, after plating, exposed to increasing doses of X-ray. After 10 d, surviving clonogenic cells were analyzed by counting cells that were able to form a cell clone or rather a colony. The *TAPIR* knockdown significantly reduces the plating efficiency and proliferation of the cells (**A**). *TAPIR-1* and *-2* knockdown leads to a significantly increased sensitization to radiation (**B**). The knockdown of *TAPIR-1* and *-2* leads to a dysregulation of DNA damage response key regulators in LNCaP-RR and to a DNA damage independent activation of the p53-DREAM signaling pathway, measured using qRT-PCR (**C**). The knockdown was validated using qRT-PCR (**D**). Data are shown as means ± s.d. *n* = 3, significance to control (SCRL): *p* ≤ 0.05 (*), *p* ≤ 0.01 (**), *p* ≤ 0.001 (***); two-sided student-*t* test.

To further explore the therapeutic potential of siRNAs against *TAPIR-1* and *-2*, additional experiments with a radiation-resistant PCa cell line (LNCaP-RR) [39,40] were carried out using a colony-formation assay (Figure 7A–D). LNCaP-RR is the only available androgen-dependent radiation-resistant cell line. The *TAPIR-1* and *-2* knockdown markedly reduced cell proliferation (colony formation), as documented by a strong reduced plating efficiency (Figure 7A). Exposing these cells to X-ray treatment, the knockdown resulted in a renewed sensitization shown by a strong decrease of the surviving cell fraction (Figure 7B). This is in line with a dysregulation of the DNA damage response key regulators in LNCaP-RR and a DNA damage independent activation of the p53-DREAM signaling pathway followed by *TAPIR-1* and *-2* knockdown.

## 3. Discussion

In search of novel cancer biomarkers using genome-wide transcriptome sequencing of a PCa screening cohort, we identified two functionally uncharacterized lncRNAs being strongly upregulated in PCa. These transcripts, which we now designated as tumor associated prostate cancer increased lncRNA (*TAPIR-1* and *-2*) are located intergenic in close proximity. Both transcripts showed remarkable diagnostic potential in tissue analysis, ranging from low to high risk PCa groups, including patients who died of the disease. The AUC value from the prostate specific antigen blood test (*PSA*; a clinical protein marker used for PCa screening) in our cohort is only 0.84, while that of *TAPIR-1* and *-2* obtained from tissue specimens amounts to ~0.94. These results illustrate the great potential of *TAPIR-1* and -2 for the development of a more sensitive and specific test. A more specific clinical PCa biomarker is the lncRNA prostate cancer antigen 3 (*PCA3*), which is overexpressed in prostate cancer cells [41]. Although the AUC of *PCA3* is somewhat lower (0.90) compared to *TAPIR-1* and *-2*, it still represents a good biomarker. However, the expression of *PCA3* was significantly lower in patients who died of their tumor. Especially these patients, who suffer from an aggressive form of the carcinoma, need to be identified by a reliable diagnostic test. According to our results, *TAPIR-1* and *-2* might be better suited to meet these requirements. Furthermore, except for the thyroid gland (in case of *TAPIR-1*), both *TAPIR* transcripts are weakly expressed in normal tissues. Overexpression was not only found in PCa but also in breast cancer tissue.

Here, we asked whether *TAPIR-1* and *-2*, in addition to their diagnostic value, also displayed therapeutic potential. By downregulating *TAPIR-1* and *-2* with siRNAs we were able to demonstrate a tumor suppressive effect. The knockdown of *TAPIR-1* and *-2* transcripts led to a cell cycle arrest, activation of p53, and downregulation of a large number of cell cycle regulators. It is known that increasing levels of p53 transcriptionally activate p21^CIP1^/*CDKN1A* protein and result in the inhibition of cyclin-dependent kinases that phosphorylate the retinoblastoma RB-related proteins p107 and p130 [28]. Hypophosphorylated p107/p130 proteins in the cell nucleus lead to the formation of the DREAM transcriptional repressor complex [24,28,42,43]. DREAM binds to E2F or CHR promoter elements of its target genes causing their transcriptional repression. The resulting p53-DREAM pathway represents the mechanism that p53 employs for indirect transcriptional downregulation of genes [24,44]. By repressing a plethora of cell cycle genes, this pathway explains how p53 can cause cell cycle arrest [24,25,43]. In contrast to the p53/*TP53* pathway, little is known about the molecular mechanisms underlying the action of lncRNAs. Through their nucleotide sequence or secondary structure, lncRNAs have been reported to bind to other cellular nucleic acids (DNA, RNA) or proteins [14,45,46]. However, the molecular events leading to the upregulation of the p53 pathway in response to siRNA knockdown of *TAPIR* transcripts are so far unknown and need to be investigated in further studies.

To test the potential of systemic *TAPIR-1* siRNA treatment to inhibit PCa growth in vivo, we established a highly aggressive PCa xenograft mouse model and successfully used PEI nanoparticle as carrier. We observed a significant decrease in tumor growth despite the large size and rapid growth of the tumor at the beginning of the treatment. Being aware of the fact that the in vivo administration of siRNAs in cancer therapy must overcome several hurdles including rapid nuclease degradation and poor delivery into target tissues, novel nanoparticle-based delivery systems, with the first siRNA drug (Patisiran) being approved in 2018, give hope to overcoming major problems [18,22,30,32]. The potential of *TAPIRs* to serve as putative therapeutic targets was underlined by a strong inhibitory effect on proliferation, also shown in radiation resistant PCa cells. The knockdown additionally results in a renewed sensitization of these cells to X-ray treatment. It has been discussed that a high cellular resistance of PCa cells to radiation goes along with an enhanced DNA-repair capability or an acquisition of a stem cell-like phenotype [47,48].

We found that the knockdown of *TAPIRs* leads to the suppression of genes essential for the DNA-damage response, as there are BRCA1 (breast cancer 1, early-onset), CHEK1 (Checkpoint kinase 1), CLSPN (Claspin), and genes encoding proteins of the Fanconi anemia pathway [49,50,51,52]. These genes are known as bona fide p53-DREAM-repressed target genes [24]. This finding helps to explain the observed phenotype. The development of a combined therapeutic approach (siRNA treatment followed by radiation) to treat a local tumor relapse after radiation therapy might be beneficial. However, this approach requires further investigation.

Should *TAPIR* be used as a target in PCa it is important to exclude that *TAPIR* siRNA treatment leads to a neurite-like phenotype. The phenotype has been described to provoke growth and cell cycle arrest [53] by deregulated expression of several cell cycle regulators, such as p27^Kip1^/*CDKN1B* members of the cyclin D protein family and CDK2 [54]. Neuroendocrine differentiation (NED) in PCa is an aggressive phenotype associated with therapy resistance. The most commonly used markers of NED are chromogranin A/CHRA) and synaptophysin/*SYP* [55]. Microarray analysis revealed that these markers were not upregulated in response to the treatment. Neither did we observe a brain-like neuronal signature in those cells. A loss of p53/*TP53* function could be a restriction on *TAPIR* siRNA application in cancer treatment. However, p53 mutations are reported in only one third of PCa patients [56] and not all of them lead to a loss of protein function. This means that *TAPIR* siRNA treatment might be effective in over two-thirds of patients.

Taken together, we here show that the knockdown of *TAPIRs* results in an upregulation of the tumor suppressor p53, tumor growth inhibition in a PCa xenograft mouse model, a reduced proliferation, and a renewed sensitization of radiation resistant cells. Thus, these data point to a significant role of *TAPIRs* in tumor occurrence and provide new approaches for diagnosis and cancer therapy.

## 4. Materials and Methods

### 4.1. Patient Characteristics

At the Department of Urology of the University Hospital Dresden (Germany) between 1995 and 2008, tissue specimens from 164 PCa patients who underwent radical prostatectomy (RPE) and non-malignant tissue samples obtained from 40 patients with benign prostate hyperplasia (BPH), undergoing surgical treatment, were included in the study. One patient of the BPH group was excluded due to wrong group assignment, resulting in a total of 203 patients (164 PCa patients and 39 BPH patients). The study was approved by the Internal Review Board at the Technische Universität Dresden (EK194092004, EK195092004), and written informed consent was obtained from all patients. Clinicopathological parameters were obtained by routine histopathological examination of the surgical specimens. Based on a Gleason score (GS) indicating the presence of regional lymph node metastases (pN) and the occurrence of death of disease (DOD), the PCa patients (*n* = 164) were stratified according to seven clinical risk groups: very low risk (V: GS < 7, pN0, *n* = 40), low risk (L: GS = 7, pN0, *n* = 40), medium risk (M, GS ≤ 7, pN1, *n* = 23), high risk survivors without lymph node infiltration (H-s, GS > 7, pN0, *n* = 20), high risk non-survivors without lymph node infiltration (H-d: GS > 7, pN0, DOD, *n* = 8), high risk survivors with lymph node infiltration (H+s: GS > 7, pN1, *n* = 19), and high risk non-survivors with lymph node infiltration (H+d: GS > 7, pN1, DOD, *n* = 14). For 52 of the high risk PCa patients, adjacent tumor-free prostate tissue samples were additionally analyzed. Information on the course of the disease, the survival of the patients, and the cause of death was obtained by contacting the general practitioners or treating urologists or from the records of the regional tumor registry.

The tissue specimens were subdivided into an exploration cohort analyzed using transcriptome-wide deep sequencing (Illumina HiSeq2000, Illumina, SD, USA), and a validation cohort was analyzed using Agilent Custom Expression SurePrint Arrays (Agilent Technologies, Waldbronn, Germany). The exploration cohort comprised 40 PCa patients, of which 40 tumor tissues and 16 tumor-adjacent tumor-free tissues were assessed, as well as non-malignant tissues from eight BPH patients as controls (in total 48 patients). The validation cohort consisted of 124 tumor tissues and 36 tumor-adjacent tumor-free tissues from PCa patients and of 31 non-malignant tissues from BPH patients (in total 155 patients).

### 4.2. Tissue Processing and RNA Isolation

Stacks of consecutive cryosections were generated from frozen prostate tissue samples and immediately transferred to 1 mL Quiazol (Qiagen, Hilden, Germany). Hematoxylin and eosin-stained cryosections flanking the stacks were evaluated by a pathologist with regard to tumor and stroma cell content. Only PCa tissue stacks, which were flanked on either side by sections containing at least 50% tumor cells were used for RNA isolation. For high risk group patients, tumor-free tissues adjacent to tumor tissue were obtained, and up to five percent tumor cells were allowed.

Total RNA was isolated using Qiazol and a miRNeasy Mini Kit on QIAcube (all from Qiagen, Hilden, Germany) followed by manual DNase I digestion (Life Technologies, Carlsbad, USA). RNA concentration was determined using a NanoDdrop 1000 (Thermo Fisher Scientific, Wilmington, USA). RNA integrity was verified on an Agilent Bioanalyzer 2100 (Agilent Technologies, Santa Clara, CA, USA). RNA samples with an RNA integrity number (RIN) of at least six were further processed.

### 4.3. Next-Generation Sequencing (NGS)

For genome-wide transcriptome analysis, isolated RNA of the exploration cohort (40 PCa patients: 24 tumor tissues and 16 matched pairs of tumor and tumor-adjacent tissues; 8 BPH patients) was used. Ribosomal RNA was depleted using the Ribo-Zero rRNA Human/Mouse/Rat removal kit (Epicentre, Madison, WI, USA) using 1 µg of total RNA as described by the manufacturers. Single indexed paired-end libraries were prepared using a ScriptSeq v2 RNA-Seq Library Preparation kit (Epicentre, Madison, WI, USA) as described by the manufacturers. For each sequencing reaction, eight independent biological replicates of an experiment were pooled after library preparation. After successful library preparation, the sequencing reaction was conducted using a HiSeq2000™ (Illumina HiSeq2000, Illumina, SD, USA) with 101 paired-end cycles.

Processing of primary sequencing data was done using workflow management [57]. CASAVA 1.8.2 was used to demultiplex FASTQ files. Adapter sequences were clipped by utilizing Cutadapt v1.6 [58]. For de novo transcript assembly, all reads were mapped to the human genome version GRCh37/hg19 with TopHat v.2.0.87 [59] setting RNA library type to fr-secondstrand and all other parameters to default values. Transcripts were assembled using cufflinks v.2.2.18 [60] with default parameters and library type specification fr-secondstrand. Assembled transcripts were compared to Gencode v17 (Ensembl 72) utilizing cuffmerge and cuffcompare [60] with default parameters in order to generate a comprehensive transcript assembly of novel and known transcripts.

For gene expression quantification, reads were mapped to the human genome version GRCh37/hg19 using segemehl v.0.1.7 [61]. Segemehl was used in split read mode (option -S) with additional parameters -H 1 -D 0 to report all alignments with no indels or mutations in the initial seed and passing the default minimal alignment accuracy. We used segemehl for expression quantification, since it outperforms TopHat in terms of sensitivity and specificity [62]. Read counts for known (Gencode v17) and novel transcripts contained in the comprehensive transcript assembly were determined using HTSeqCount v0.6.0 [63] with parameters mode intersection-strict, stranded yes, idattr gene_id, and type exon.

Differential gene expression between tumor and control samples was assessed using the statistical software package R v3.2. [64] and the Bioconductor package DeSeq2 v1.81 [65]. Raw read counts were normalized and variance stabilized (vst transformation). False discovery rate (FDR) was controlled using Benjamini-Hochberg adjustment [66]. Discrimination between BPH and tumor specimens for each lncRNA (*TAPIR-1* und *-2*) was evaluated using receiver operating characteristic (ROC) curves and area under the curve (AUC) using R package pROC v1.10.0 [67]. Confidence intervals for AUC and ROC were estimated using 2000 bootstrap replicates (pROC). Data were visualized via UCSC Genome Browser [68] or Integrative Genomics Viewer [69].

### 4.4. Agilent Custom Expression Microarray Processing

For gene expression profiling, total RNA was extracted from prostate specimens of the validation cohort (164 PCa patients, with tumor-adjacent tissues for 52 PCa patients, and 39 BPH patients). Note, in our in vitro study, only patients who had not undergone any androgen deprivation therapy prior to surgery were included. We focused our investigations on androgen-dependent PCa. 200 ng of total RNA was labeled using a Quick Amp Labeling Kit one color (Agilent Technologies) with a custom N6T7 random primer, following the manufacturer’s instructions. RNA was hybridized to Agilent Sureprint G3 Custom Exon 4 × 180k microarray (Design-ID: 058029, GEO platform ID GPL26898). Array read-out was performed with an Agilent Microarray Scanner System G2565CA as described by the manufacturers.

Differential expression analysis in the validation cohort was only conducted for patients not included in the exploration cohort (124 PCa patients, with tumor-adjacent tissues for 36 PCa patients, and 31 BPH patients). Differentially expressed probes were identified by using R v3.2.2 [64] and the Bioconductor package limma v3.24.15 [70]. Quality control of arrays was performed by checking distribution of “bright corner” and “dark corner” probes and relative spike-in concentration versus normalized signal. The controls confirmed the high quality of the results and consequently all microarrays were included in downstream analyses. To retrieve a set of probes mapping to unique genomic positions in hg19 we used BLAT v35 [71] with the parameter –minIdentity = 95 allowing probes spanning splice sites to be detected. All probes mapping to more than one distinct genomic region were discarded. Normalization between arrays was done using quantile normalization [72]. In order to reduce the number of univariate *t*-tests, nonspecific filtering was applied as follows: the expression of a probe must be larger than background expression in at least 20 arrays. Background expression was defined by the mean intensity plus three times the standard deviation (s.d.) of negative control spots (Agilent’s 3xSLv spots). In addition, a probe must exhibit a nonspecific change of expression, which was assessed using an interquartile range of at least 0.5. Finally, a linear model to detect expression variation between BPH and tumor specimens adjusted for array processing batches was fitted using limma v3.24.15, and reliable variance estimates were obtained using the empirical Bayes moderated t-statistics test. False discovery rate (FDR) was controlled using Benjamini-Hochberg adjustment [66]. Discrimination between BPH and tumor specimens for each lncRNA (*TAPIR-1* und *-2*) was evaluated using ROC curves and AUC using R package pROC v1.10.0 [67]. Confidence intervals for AUC and ROC were estimated using 2000 bootstrap replicates (pROC).

### 4.5. Coding Potential Prediction

To determine the coding potential probability, we used the Coding-Potential Assessment Tool (CPAT; http://lilab.research.bcm.edu/cpat/index.php), an alignment-free program which uses logistic regression to distinguish between coding and noncoding transcripts on the basis of four sequence features (ORF size, ORF coverage, Fickett TESTCODE statistic, and hexamer usage bias). A resulting coding probability below 0.364 (cutoff) denotes non-coding transcripts with high accuracy (0.967) [23].

### 4.6. Design and Synthesis of Small Interfering RNAs

Stealth RNAi^TM^ siRNA Duplex (Invitrogen, cat#10620312) were designed using a siRNA target finder tool (Invitrogen, Block-iT^TM^ RNAi Designer), to silence the following target transcripts: *TAPIR-1* (GeneBank accession no. AC141930.1, ENSG00000228613 Homo sapiens: GRCh37.p13 (GCF_000001405.25) Chr2 (NC_000002.11): 1,550,437-1,623,885) and *TAPIR-2* (GeneBank accession no. AC144450.2 ENSG00000203635 Homo sapiens: GRCh38.p12 (GCF_000001405.38) Chr2 (NC_000002.12): 1,620,510-1,625,419). Four *TAPIR* specific siRNAs were used in knockdown studies (si-A1, si-B1, si-A2, si-B2). A BLAST search (http://www.ncbi.nlm.nih.gov/BLAST) for the selected siRNA sequences was carried out to exclude any alignment with other sequences in the human genome. As negative control, Stealth RNAi^TM^ Negative Control Medium GC Duplex (Invitrogen, cat# 12935-300) with no significant homology to any known human mRNA was used for subsequent normalization. To silence p53/TP53 and p21^CIP1^/CDKN1A expression, bona fide p53-siRNA (cat# 1299001 VHS40366) and p21^CIP1^*/CDKN1A*-siRNA (cat# 1299001 VHS40209) purchased from Invitrogen were used. Each 2 × 10^6^ cell was transfected with 200 pmoles of Stealth^TM^ siRNA. All siRNAs used in knockdown experiments are listed in Table 1. Transfection was carried out using a NEON™ kit and the microporator MP100 Digitalbio (LIFE Technologies), according to the manufacturer’s instructions. Two pulses of 1250 V and 20 ms were applied.

### 4.7. Cell Lines, Cell Culture, and Tissue Samples

The PCa cell lines LNCaP (derived from metastatic site—left supraclavicular lymph node) and DU145 (derived from metastatic site—brain) was obtained from ATCC and grown in RPMI-1640 medium (Gibco, Life Technologies, Carlsbad, USA), supplemented with 10% fetal bovine serum (FBS; gibco, 10270-106). The PCa cell line MDA-PCa2b (derived from metastatic site—bone) was obtained from ATCC and cultured in F-12K medium (Gibco, Life Technologies, Carlsbad, USA), supplemented with 20% none heat-inactivated fetal bovine serum (FBS; Gibco, Life Technologies, Carlsbad, USA), 25 ng/mL cholera toxin (Sigma-Aldrich, St. Louis, MO, USA), 10 ng/mL mouse Epidermal Growth Factor (Sigma), 0.005 mM phosphoethanolamine (Sigma), 100 pg/mL hydrocortisone (Sigma), 45 nM sodium selenite (Sigma), and 0.005 mg/mL human recombinant insulin (Sigma). The radioresistant properties of LNCaP RR cells were validated prior to experiments. The cell lines were cultured in a humid atmosphere at 37 °C and 5% CO_2_. Every batch tested negative for mycoplasma contamination. The cell lines were genotyped using microsatellite polymorphism analyses.

### 4.8. Microarray Analysis Following TAPIR Knockdown

Knockdown studies were performed in LNCaP cells by targeting either *TAPIR-1* (si-A1), *TAPIR-2* (si-A2), or nothing, in case of scrambled siRNA (si-SCRL). Total RNA was isolated from cells with TRIzol (ambion), following the manufacturer’s protocol. For each siRNA type, four biological replicates were prepared for two temporal conditions: 24 h and 48 h after the treatment. The arrays were of type Agilent SurePrint G3 Human Gene Expression 8 ×  60K v2 (24h) (design ID Agilent: G4851B) and v3 (48 h) (design ID Agilent: G4851C). The arrays were scanned with a microarray scanner (Agilent Technologies). Differentially expressed probes were identified using R v3.4.3 [64] and the Bioconductor package limma v3.34.8 [70]. Quality control of arrays were performed by checking the distribution of “bright corner” and “dark corner” probes and unsupervised clustering of raw intensities. One replicate of si-A2 at 48 h had to be removed from downstream analyses due to too low foreground intensities. To retrieve a set of probes mapping to unique genomic positions in hg38 we used BLAT v35 [71] with the parameter –minIdentity = 95 allowing probes spanning splice sites to be detected for all arrays. All probes mapping to more than one distinct genomic region were discarded. Remaining probes were annotated by known genes using Gencode v27. Normalization between arrays was done using quantile normalization [72], and batch effects were considered as additive covariates in the linear model. In order to reduce the number of univariate *t*-tests, nonspecific filtering was applied as follows: the expression of a probe had to be larger than the background expression in at least three arrays. Background expression was defined by the mean intensity plus three times the s.d. deviation of negative control spots (Agilent’s 3xSLv spots). In addition, a probe must exhibit a nonspecific change of expression, which was assessed using an interquartile range of at least 0.5. Finally, a linear model was fitted using limma to assess significant expression changes for the contrasts si-A1 versus si-SCRL and si-A2 versus si-SCRL, respectively. Reliable variance estimates were obtained using an empirical Bayes moderated t-statistics test. FDR was controlled using Benjamini-Hochberg adjustment [66]. All genes with a log2 fold change ≥0.5 and FDR < 0.01 were classified as being significantly regulated upon *TAPIR-1* or *TAPIR-2* knockdown.

Functional analysis among all significantly regulated genes compared to the background set of all genes covered by at least one probe of the microarray was conducted for gene ontology (GO) categories, as well as for curated pathway annotations (Kyoto Encyclopedia of Genes and Genomes (KEGG) [73] and Reactome [74]). Gene ontology enrichment analyses were realized by using the Bioconductor package topGO v2.32.0 [75]. To detect significantly enriched GO categories, we applied Fisher’s exact test using the classic algorithm. P-values were adjusted for multiple testing (Benjamini-Hochberg correction). KEGG pathway enrichment analysis was conducted with the Bioconductor package graphite v1.26.3 [76] in combination with SPIA v2.32.0 [77] In detail, KEGG pathways were tested with the function runSPIA() of the graphite package, an algorithm respecting topology dependent expression changes of genes in the same pathway. The global *p*-values were adjusted by applying the FDR algorithm according to Benjamini and Yekutieli [78] (pGFdr <0.05). KEGG pathway enrichment was complemented with the Reactome database to combine evidence from two different pathway databases. Overrepresentation of genes associated to pathways as annotated in Reactome were identified by a hypergeometric test using the R package ReactomePA v1.24.0 [79] with the associated Bioconductor reactome.db v1.64.0 [80]. The false discovery rate (FDR) of the Reactome pathways was controlled using Benjamini-Hochberg adjustment [66].

### 4.9. Assessment of Pan-Tissue and Pan-Cancer TAPIR Expression

The Genotype-Tissue Expression (GTEx; https://gtexportal.org/home/) project [81] provided public transcriptome sequencing gene data analysis from 53 normal tissue types (8555 samples; 570 donors), released as V7 version (dbGaP Accession phs000424.v7.p2). Gene expression for *TAPIR-1* (AC144450.1, NSG00000228613.1) and gene expression for *TAPIR-2* (AC144450.2, ENSG00000203635.2) was plotted.

### 4.10. Isolation of RNA, cDNA Synthesis, and PCR Analysis

Total RNA was isolated from cell lines and xenograft tissue with a TRIzol (Ambion) or RNaesy Kit (Qiagen), following the manufacturer’s protocol. RNA was DNase-digested using a TURBO-DNA-free kit (Ambion). Reverse transcription of RNA was conducted using a RevertAid RT Kit (Thermo Fisher scientific) or PrimeScipt RT Kit (TaKaRa, Beijing, China). Analysis of cDNA was performed using either GoTaq qPCR Master Mix (Promega) as described by the manufacturer using a CFX Connect Real-Time System (Bio-Rad), StepOne Real-Time PCR System (Applied Biosystems), or standard PCR. Primers are listed in Table 2. Each (RT)-qPCR reaction was performed in at least three independent biological replicates. Data are shown as means (±s.d.). For indicated experiments, a two-sided *t*-test was used to assess statistical significance. The ∆Ct value was determined by subtracting the Ct value of housekeeper Ct value (*GAPDH*, *β-actin*) from the target Ct value (*AURKA*, *Survivin/BIRC-5*, MYBL2, *CCNB1*, *CCNB2*, *CCNE1*, *CDC25C*, *E2F1*, *FOXM1*, *HISTH1B*, *KIF23*, *MKI67*, *p21^CIP1^/CDKN1A*, *AR*, *TAPIR-1*, and *TAPIR-2*).

### 4.11. Xcelligence

The xCELLigence^®^, (Roche, Basel, Switzerland) real-time cell analysis (RTCA) instrument was used to non-invasively monitor cell status including cell number, shape/size, and attachment. Each 1 ×  10^5^ cell, transfected with control or *TAPIR* siRNA, were seeded and cultured per well of an electronic 96-well microtiter plate (E-Plate^®^, Roche, Basel, Switzerland) with gold microelectrodes embedded in the bottom. The impedance value was measured, plotted as a unit-less parameter called “Cell Index”, and increases as cells proliferate. The continuous acquisition of impedance data for each well of an E-Plate enables the generation of real-time proliferation curves. The observation endpoint was 6 d after transfection. Each experiment was performed as technical quadruplicate and biological triplicate.

### 4.12. IncuCyte ZOOM™

LNCaP cells were cultured and transfected with siRNAs as described above. Cells were removed from the monolayer using Trypsin (SERVA) in phosphate buffered saline (PBS), resuspended in medium and seeded at a density of 4 ×  10^5^ cells per well in 96-well ImageLock plates (Satorius). After seeding, cells were grown for 6 d and monitored using the automated IncuCyte ZOOM live cell imaging system (Satorius), hourly. Images were analyzed using the IncuCyte ZOOM’s Confluence Processing analysis tool (Basic Analyzer), which calculates and provides cell metrics.

### 4.13. Cell Cycle Analysis

For cell cycle analysis, 5 ×  10^5^ cells were fixed 72 h after transfection in 4 mL ice cold ethanol (80%) for 20 min at −20 °C. DNA content was labelled with propidium iodide (PI) by adding 100 µL PBS, 100 µL FACS buffer (0.1 M sodium acetate, 0.5 M EDTA), 50 µL RNase (Qiagen, 1 mg/mL FACS buffer), 20 µL Triton-X 100 (0.2% in FACS buffer) and 10 µL propidium iodide (1 mg/mL) for 30 min at 37 °C and measured in the FACSCalibur flow cytometer (BD Biosciences). Data represent the mean ± s.d. of a minimum of three biological replicates.

### 4.14. Westernblot

After transfection, each 1 ×  10^6^ cells were seeded into 6 cm diameter cell culture dishes and cultured for 48 h and 72 h. Subsequently, cells were washed with PBS. Protein lysates were prepared by adding 200 µL Ripa lysis buffer (supplemented with 1 mM NaVanadat, 1 mM PMSF, 1 mM Aprotenin, 1 mM Leupeptin, 1 mM Pepstatin), scraping, incubation on ice (30 min), and sonication (30 s, 3 strokes) followed by centrifugation for 10 min, 4 °C at 13,000 rpm. The concentration of protein in a solution was measured by Bradford protein assay [82]. SDS-PAGE and western blot were performed following standard protocols as described earlier [83]. The following antibodies were applied for protein detection: anti-β-actin (Sigma Aldrich, mouse mAb, clone AC-15, #A5441, dilution 1:5000), anti-CDC25C (Santa Cruz Biotech, mouse mAb, H6, #sc-13138, dilution 1:500), anti-cyclin B2/CCNB2 (Santa Cruz Biotech, mouse mAb, A-2, #sc-28303, dilution 1:500), anti-KIF23/MKLP-1 (Santa Cruz Biotech, mouse mAb, 24, #sc-136473, dilution 1:500), anti- p21^CIP1^/CDKN1A (Calbiochem, mouse mAb, Ab-1, EA10, # OP64, dilution 1:1000), anti-p53 (Calbiochem mouse mAb, Ab-6, DO-1, #OP43, dilution 1:2000), and anti-Survivin (Cell Signaling, rabbit mAb, 71G4B7, #2808, dilution 1:1000). The monoclonal B-Myb LX015.1 antibody (hybridoma media 1:2) was a kind gift from Roger Watson [84]. Uncropped western blot images are shown in Figure S13.

### 4.15. p53 Pathway Knockdown Experiments

LNCaP (each 1 ×  10^6^ cells) were transfected with si-SCRL; si-A1; si-SCRL and si-53; si-SCRL and si-p21^CIP1^; si-A1 and si-p21^CIP1^; si-A1 and si-p53; si-A1, si-p21^CIP1^, and si-p53, as described above, and cultured for 48 h and 72 h at 37 °C in a humid atmosphere. Western blot and qPCR analysis were carried out as described above.

### 4.16. ViewRNA™ In Situ Hybridization (ISH)

In situ hybridization was performed using the ViewRNA^TM^ ISH Tissue 2-Plex Assay Kit (Affymetrix/invitrogen, Santa Clara, USA, # QVT0012) following the manufacturer’s protocol. Slides from high grade PCa tissue were baked at 60 °C for 60 min and deparaffinized in xylene twice for 10 min. Next, slides were incubated twice in 100% ethanol for 10 min and air dried. To allow target accessibility slides were incubated in 1×  pretreatment solution at 90 °C for 10 min, washed in ddH_2_O, and treated with protease solution (1:100 in 1×  PBS) at 40 °C for 20 min. After fixation in 4% buffered paraformaldehyde (PFA) for 3.5 min at room temperature (RT), slides were washed in PBS. Target probe sets (1:40 in Probe Set Diluent QT) designed against PSA, *TAPIR-1*, *TAPIR-2*, and *β-galactosidase gene* (*lacZ*) as a negative control (Affymetrix, Santa Clara, USA; Cat No VA1-10381, VA1-19031, VA1-19032, VF1-12414) were added to the slides and incubated at 40 °C for 2 h. Slides were washed in wash buffer and stored in storage buffer at RT for up to 24 h subsequently. Next, slides were washed in wash buffer, and signal amplification was achieved via a series of sequential hybridization steps. PreAmplifier Mix QT was added to the slides and incubated at 40 °C for 25 min. After washing the slides in wash buffer, Amplifier Mix QT was added to the slides followed by incubation at 40 °C for 25 min. Again slides were washed in wash buffer and Label Probe-AP (Label Probe 6-AP 1:1000 or Label Probe 1-AP 1:250 in Label Probe Diluent, according to target probe type) was added to slides and incubated at 40 °C for 25 min. Afterwards, slides were washed in wash buffer, fast blue substrate was added, and the reaction took place at RT in the dark for 30 min. Slides were successively washed in wash buffer, PBS and ddH_2_O. Counterstaining took place for 3 min using nuclear fast red aluminium sulphate solution (Carl Roth; N069). Finally, slides were rinsed in H_2_O, dehydrated by ascending alcohol series and mounted with Entellan^®^new (Merck, Darmstadt, Germany). Tissue slides were scanned and digitalized using an Ultra Fast Scanner (digital pathology slide scanner; PHILIPS).

### 4.17. Mouse Xenograft Model and Therapeutic In Vivo siRNA Treatment

Six‒eight-week-old immunodeficient mice (NOD/SCID/IL2r gamma (null), Jackson Laboratory) were kept in cages with rodent chow (ssniff) and water available ad libitum. Animal studies were performed according to the national regulations and approved by the local authorities, ethical code number 38/17, Landesdirektion Sachsen, Germany. For the generation of tumor xenografts, 5 × 10^6^ MDA-PCa2b cells in 150 µL PBS were injected subcutaneously into both mouse flanks, after shaving the injection region. When the tumors were established after 5 weeks, mice were randomized into treatment (PEI/siA1) and control groups (PEI/siCtrl), with *n* = 13 tumors per group. Polymeric nanoparticles (PEI/siRNA complexes) based on low molecular weight branched polyethylenimine PEI F25-LMW [30] were prepared in HN buffer (10 mM HEPES, 150 mM NaCl), as described previously [32]. Mice were treated at the time points indicated in Figure 6 using an intraperitoneal injection of PEI F25-LMW/siRNA complexes containing 10 µg siRNA (~0.4 mg siRNA/kg body weight; stealth scrambled mock control; si-Neg.Ctrl. 5′-CCCUAACGAGGGUUA-3′ [85] or siRNA-*TAPIR-1*; si-A1: 5′-CGTCTGTCTGGAGTTTCCGCTTCAT-3′) in 150 µL HN buffer. Tumor volumes were measured in all three dimensions (length × width × height). Upon termination of the experiment, mice were sacrificed, and tumors were immediately removed. Pieces of the tumor were directly snap-frozen for RNA analysis and protein preparation. The tumor tissue was obtained from xenograft mice, using a scalpel, and cut with an autoclaved standard razor blade into 5-mm cubes. Tissue specimens were stored at −80 °C and cut into 15 µm slices using a cryotome™. For each tumor, 20 tissue slices were pooled, homogenized using a ball mixer mile (PRECELLYS^®^ 24; Bertin Instruments); 3 balls, 3 min at 6000 rpm), and total RNA was extracted as described above. Quantification of *TAPIR* was done using qPCR as described above.

### 4.18. Clonogenic Cell Survival Assay

Cells were plated at a density of 500–1000 cells/well depending on the cell line in 6-well plates and irradiated with doses of 0, 2, and 4 Gy of 200 kV X-rays (Yxlon Y.TU 320; dose rate 1.3 Gy/minute). The absorbed dose was measured using a Duplex dosimeter (PTW). After 10 days, the colonies were fixed with 10% formaldehyde (VWR) and stained with 0.05% crystal violet (Sigma-Aldrich). Colonies containing >50 cells were counted using a stereo microscope (Zeiss). The plating efficiency (PE) was determined as the ratio between the generated colonies and the number of cells plated. The surviving fraction (SF) was calculated as the PE from the irradiated cells divided by the PE from the nonirradiated control.

### 4.19. Statistical Analysis

Experimental data are represented unless otherwise stated in the methods or legends as the mean ± s.d. of a minimum of three biological replicates and were compared using Student’s *t*-test. Significant *p* values are indicated with asterisks as follows: * *p* < 0.05, ** *p* < 0.01, and *** *p* < 0.001.

### 4.20. Data Availability

All gene expression studies are accessible from the Gene Expression Omnibus (GEO) database. NGS of the exploration cohort of PCa and BPH tissue specimens: GSE134073 [reviewer access token: crgtacqetjudhyn], Agilent custom expression microarray of PCa and BPH tissue specimens (covering all patients included in exploration and validation cohort): GSE134051 [reviewer access token: gtcvummelhmnryv], and Agilent expression microarray analysis following *TAPIR* knockdown: GSE134015 [reviewer access token: idqhiokwdtwpbev].

## 5. Conclusions

In this study, we discovered two lncRNA transcripts, designated here as *TAPIR-1* and *-2*, which are strongly upregulated in prostate tissue specimens of patients suffering from PCa. The *TAPIR* expression allows a remarkably precise diagnostic discrimination between PCa tumor and benign control samples (AUC = 0.94), as shown for a cohort comprising samples from more than 200 patients. Therefore, the development of a diagnostic PCa test is in progress.

Furthermore, we show that *TAPIR* lncRNAs may also serve as promising therapeutic targets. The siRNA-based knockdown of *TAPIR-1* and *-2* results in an upregulation of the tumor suppressor p53 protein, which activates the p53/TP53-DREAM pathway. This mechanism leads to the downregulation/inhibition of CDKs and many other cell cycle regulating proteins, which finally results in cell cycle arrest. Accordingly, in a preclinical PCa xenograft model in mice, the systemic application of nanoparticles loaded with siRNA targeting *TAPIR-1* significantly reduced tumor growth in vivo. Furthermore, the activation of the p53-DREAM pathway leads to suppression of essential key regulators of the DNA-damage response, as there are *BRCA1*, *CHEK1*, *CLSPN*, and genes encoding proteins of the Fanconi anemia pathway. Therefore, we studied the effect of *TAPIR* siRNA when treating radiation therapy-resistant tumor cells. In addition to a strong proliferation inhibition, the knockdown leads to an impressive renewed sensitization of these cells to X-ray treatment. The influence of castration resistance and chemoresistance must be investigated in follow up studies.

## 6. Patents

The following patents have resulted from the work reported in this manuscript: RNA-biomarkers for diagnosis of prostate cancer EP3077537A1 (EU) and EP3077537B1 (US).

## Figures and Tables

**Table 1:** List of siRNAs used in the study

RNA Stealth siOligos and siRNA			Application	Target
*TAPIR-1*	si-A1	5′-CGTCTGTCTGGAGTTTCCGCTTCAT-3′	*TAPIR-1* knockdown	Exon 3
*TAPIR-1*	si-B1	5′-CCAGGGAGCACACTTTATTAGGAAA-3′	*TAPIR-1* knockdown	Exon 3
*TAPIR-2*	si-A2	5′-GGTCAGCGTCTCGTTCACCTCTTTA-3′	*TAPIR-2* knockdown	Exon 2
*TAPIR-2*	si-B2	5′-CCGTGACAGTTTGAGTTGAGGACAT-3′	*TAPIR-2* knockdown	Exon 2
p53/TP53	si-p53	Invitrogen cat# 1299001 VHS40366	p53 knockdown	n.d.
p21^CIP1^/CDKN1A	si-p21^CIP1^	Invitrogen, cat# 1299001 VHS40209	p21^CIP1^ knockdown	n.d.
AR	si-AR	Dharmacon, cat# L-003400-00-0005	AR knockdown	n.d.
siRNA control (medium GC)	si-SCRL	Invitrogen, cat# 12935-300	negative control	none

(n.d.—not described by company).

**Table 2:** List of oligonucleotides used in the study.

Target	Strand	Sequence	Application	Product Size (bp)
GAPDH	For Rev	GTCAGTGGTGGACCTGACCT AGGGGAGATTCAGTGTGGTG	housekeeper, qPCR, normalization	395
ß-actin	For Rev	TCGTGCGTGACATTAAGGAGAA AGCAGCCGTGGCCATCT	housekeeper, qPCR, normalization	71
*TAPIR-1* (Ex3)	For Rev	TTCATAGACCGTGCTTCCGT TGTGAACCTGGAGCTTTCCT	qPCR TAPIR-1, RNA expression	271
*TAPIR-1* (Ex2-3)	For Rev	GGGACAACTCGAAGCAAAGG CTACAGAATGCGTGGTGTGG	qPCR TAPIR-1, RNA expression	389
*TAPIR-1* (Ex1-2)	For Rev	TCTCAAAGCAGGCTCCATGA CGTCTGCTTCGAGTCTTCCT	qPCR TAPIR-1, RNA expression	249
*TAPIR-2* (Ex1-2)	For Rev	GACTTCACCACTCACCAGGA AGGGACCCGTCTTATGTGAC	qPCR TAPIR-2, RNA expression	237
AURKA	For Rev	TTGAACACCCCTGGATCACA AGCGTTCTAGATTGAGGGCA	Validation of microarray, qPCR, RNA expression	280
CCNB1	For Rev	TGGTGCACTTTCCTCCTTCT TTAGCATGCTTCGATGTGGC	Validation of microarray, qPCR, RNA expression	420
CCNB2	For Rev	GCTGAACTCAAAAGCCGTCA ACTGGTCTGAGAAGAGGTTTCA	Validation of microarray, qPCR, RNA expression	306
p21CIP1/ *CDKN1A*	For Rev	TGTCTTGTACCCTTGTGCCT AAGATGTAGAGCGGGCCTTT	Validation of microarray, qPCR, RNA expression	480
E2F1	For Rev	CTCTAACTGCACTTTCGGCC CCTCCTCCCCTTTGCTGATT	Validation of microarray, qPCR, RNA expression	360
HIST1H1B	For Rev	ACTCCGAAGAAGGCGAAGAA GCTTTAGGTTTTGCTGCTTTGG	Validation of microarray, qPCR, RNA expression	321
Survivin/ BIRC5	For Rev	TGATGAGAGAATGGAGACAGAG ACAGCAGTGGCAAAAGGAG	Validation of microarray, qPCR, RNA expression	223
MYBL2	For Rev	CAGCAATGCCAGTACAGGTG CTCAGGGTTGAGGTGGTTGT	Validation of microarray qPCR	198
CCNE1	For Rev	GCAGGATCCAGATGAAGAAATG GGGTCTGCACAGACTGCAT	Validation of microarray qPCR	96
CDC25C	For Rev	CTCAGATGCTGGAGGAAGAATC GGGACGATGGGCTTCTTCAG	Validation of microarray qPCR	283
FOXM1	For Rev	CGTGGATTGAGGACCACTTT GGCTTAAACACCTGGTCCAA	Validation of microarray qPCR	187
MKI67	For Rev	CCAGCTTCCTGTTGTGTCAA AGCCGTACAGGCTCATCAAT	Validation of microarray qPCR	129
KIF23	For Rev	GTAGAGTGGCAGCCAAACAGC CTGATCAGGTTGAAAGAGTAAAGGC	Validation of microarray qPCR	197
AR	For Rev	GCCAGCAGAAATGATTG AGCCTCTCCTTCCTCCTG	qPCR AR, RNA expression	150
BRCA1	For Rev	CAGCTTGACACAGGTTTG GGATTTTCGGGTTCACTC	qPCR, RNA expression	155
ATR	For Rev	AACCTCCGTGATGTTGCTTG AATGACAGGAGGGAGTTGCT	qPCR, RNA expression	164
CHEK1	For Rev	TCAGACTTTGGCTTGGCAAC GGTTGGTCCCATGGCAATTC	qPCR, RNA expression	194

## Supplementary Materials

The following are available online at https://www.mdpi.com/2072-6694/12/5/1122/s1, Figure S1: Expression of *TAPIR-1* and *-2* in prostate tissue of an exploration cohort of BPH and PCa patients, Figure S2: Expression patterns of PCA3 in the validation cohort related to clinical risk classification, Figure S3: Visualization of TAPIR transcripts expression in FFPE PCa sections by ViewRNA in situ hybridization analysis, Figure S4: *TAPIR-1* and *-2* expression is low in normal tissue, Figure S5: *TAPIR-1* and *-2* overexpression is restricted to tumor tissue, Figure S6: TAPIRs knockdown with 4 different TAPIR-directed siRNAs in LNCaP cells, Figure S7: Comparison between primary tumors and cell lines and siRNA knockdown based phenotype of TAPIR lncRNAs positive and negative cells, Figure S8: Expression pattern of *TAPIR-1* and *-2* after androgen receptor knockdown, Figure S9: Enlarged graph of the ReactomePA pathway analysis of Figure 4A, Figure S10: Increase in p53 levels and downregulation of cell cycle and DNA repair control genes following *TAPIR-1* knockdown, Figure S11: Enlarged graph of the ReactomePA pathway analysis of Figure S10, Figure S12: Validation of cell cycle regulating target genes after *TAPIR-1* and *-2* knockdown by qPCR, Figure S13: Gene expression alterations after knockdown of p53 or *TAPIR-1* 48 h post siRNA transfection, Figure S14: Gene expression alterations after knockdown of p53 or *TAPIR-1* 72 h post siRNA transfection, Figure S15: Uncropped western blot images, Table S1: Coding potential prediction of *TAPIR-1* and *-2* represent non-coding used Coding-Potential Assessment Tool (CPAT), Table S2: Biological process; GO term enrichment analyses of siRNA knockdown experiments in LNCaP cells, Table S3: Molecular function; GO term enrichment analyses of siRNA knockdown experiments in LNCaP cells, Table S4: Cellular compartment; GO term enrichment analyses of siRNA knockdown experiments in LNCaP cells, Excel file S1: 24h_*TAPIR-1*-scramble, Excel file S2: 24h_*TAPIR-2*-scramble.
