# Supplementary material for: The Role of lncRNAs TAPIR-1 and -2 as Diagnostic Markers and Potential Therapeutic Targets in Prostate Cancer
Source: Cancers (Basel). 2020 Apr 30;12(5):1122. doi: 10.3390/cancers12051122 (PMC7280983; doi:10.3390/cancers12051122)
Supplement: Supplementary file 1 [file cancers-12-01122-s001.zip › SUPP/cancers-740908-supp-final.pdf]

Supplementary Materials: The Role of lncRNAs TAPIR-1 and -2 as Diagnostic Markers and Potential Therapeutic Targets in Prostate Cancer.

Maik Friedrich, Karolin Wiedemann, Kristin Reiche, Sven-Holger Puppel, Gabriele Pfeifer, Ivonne Zipfel, Stefanie Binder, Ulrike Köhl, Gerd A. Müller, Kurt Engeland, Achim Aigner, Susanne Füssel, Michael Fröhner, Claudia Peitzsch, Anna Dubrovska, Michael Rade, Sabina Christ, Stephan Schreiber, Jörg Hackermüller, Jörg Lehmann, Marieta I. Toma, Michael H. Muders, Ulrich Sommer, Gustavo B. Baretton, Manfred Wirth and Friedemann Horn

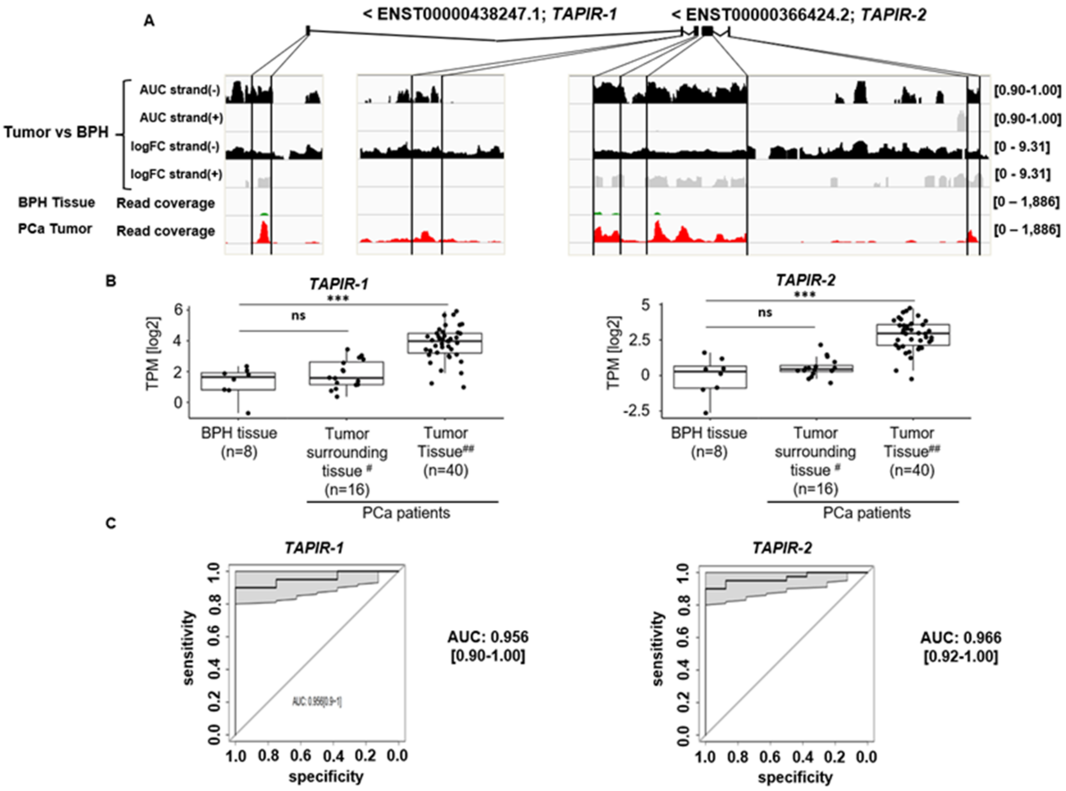

Figure S1. Expression of *TAPIR-1* and *-2* in prostate tissue of an exploration cohort of BPH- and PCa patients. RNA expression detected by transcriptome-wide NGS analysis of the exploration cohort comprising tissue specimens from 40 PCa patients (40 tumor tissues and 16 paired tumor-adjacent tumor-free tissues) and 8 BPH patients. (A) Area under the ROC curve to discriminate PCa tissue from BPH tissue (AUC; for both strands), logarithmic fold change (logFC; for both strands) and read coverage of a representative PCa and a BPH patient. Values for AUC (range 0.9-1.00), logFC (0-9.31) and read coverage (0-1.886) are shown in squared brackets. (B-C) Box plots and area under the ROC (AUC) analysis of *TAPIR-1* and *-2* are shown. ROC analysis for the TAPIR-1 and -2 revealed PCa diagnostic AUC values of 0.96 [0.90-1.00] and 0.97 [0.92-1.00], respectively, FDR≤0.001 (***). Non-significant = ns.

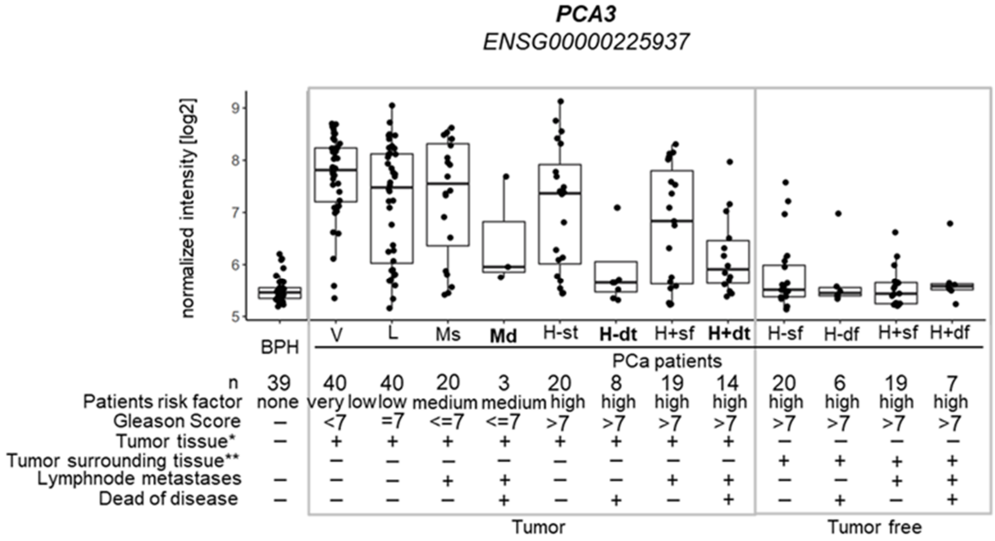

Figure S2. Expression patterns of PCA3 in the validation cohort related to clinical risk classification. Expression patterns of *PCA3* determined by microarray analysis is shown related to clinical risk classification. Normalized expression intensity [log2] was plotted against subgroups based on clinical data sets: patient risk factor (none, very low, low, and high); Gleason Score (none, =7, ≤7, >7); tumor tissue (-/+), verified tumor cell content >60% for tumor tissue (denoted with *; -/+); matched tumor adjacent tissue (-/+), verified tumor cell content 0-5% for matched tumor surrounding tissue (denoted with **; -/+); lymph node metastases (-/+), died of disease (-/+). Groups are defined as follows: BPH, PCa-risk groups: V= very low; L=low; Ms=medium, with lymph node metastases; Md=medium, with lymph node metastases and death because of disease (DoD); Tumor tissue (t): H-st= high, without metastases; H-dt= high, without metastases and DoD; H+st= high with lymph node metastases; and H+dt= high, with metastases and DoD; Matched tumor (free) adjacent tissue (f): H-sf= high, without metastases; H-df= high, without metastases and DoD; H+sf= high with lymph node.

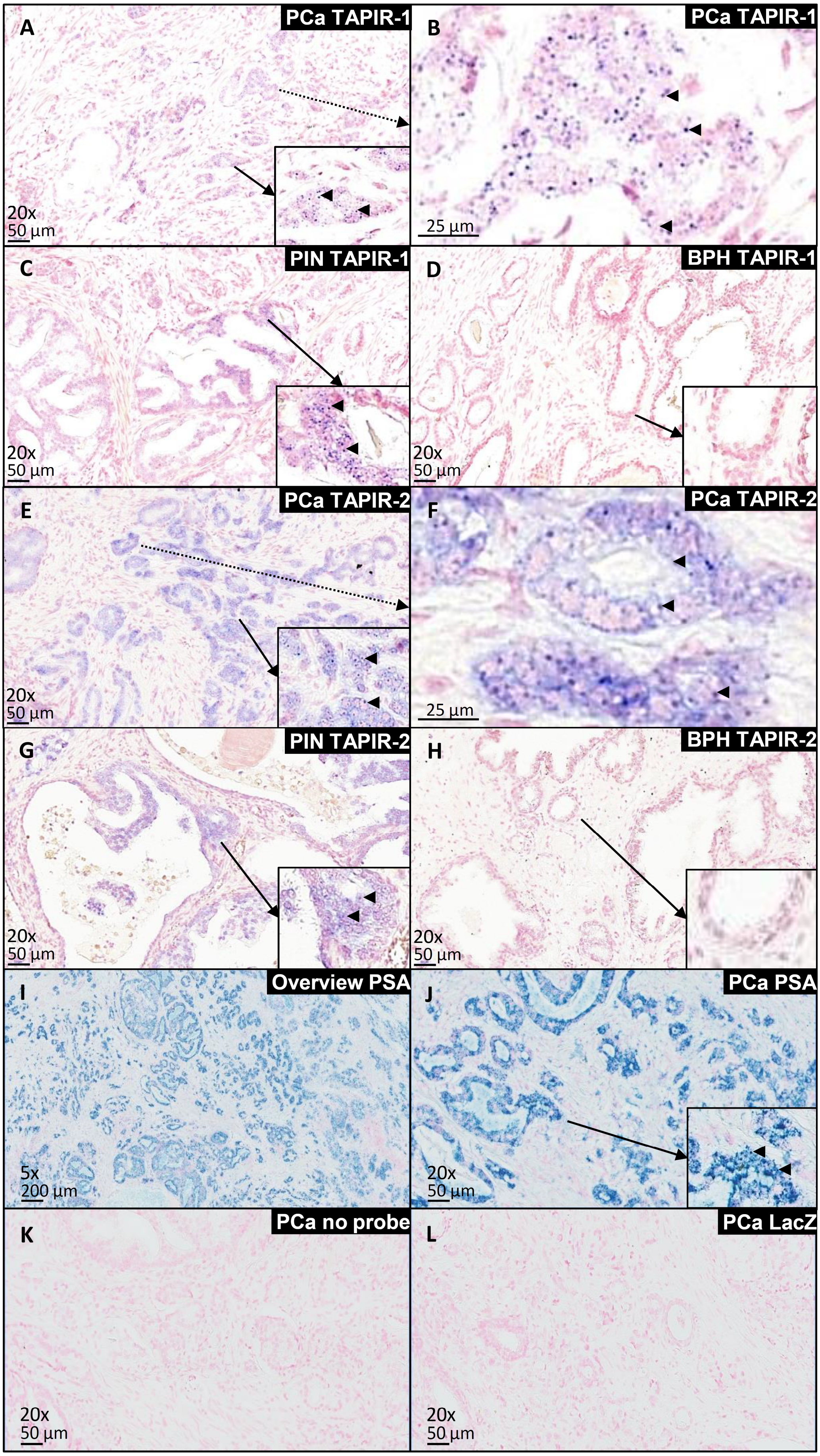
A
PCa TAPIR-1
20x
50 µm
B
PCa TAPIR-1
25 µm
C
PIN TAPIR-1
20x
50 µm
D
BPH TAPIR-1
20x
50 µm
E
PCa TAPIR-2
20x
50 µm
F
PCa TAPIR-2
25 µm
G
PIN TAPIR-2
20x
50 µm
H
BPH TAPIR-2
20x
50 µm
I
Overview PSA
5x
200 µm
J
PCa PSA
20x
50 µm
K
PCa no probe
20x
50 µm
L
PCa LacZ
20x
50 µm

Figure S3. Visualization of *TAPIR* transcripts expression in FFPE PCa sections by ViewRNA in situ hybridization analysis. In situ hybridization was performed using the ViewRNA™ technology, following the manufacturer's protocol. Large cut sections of a selected high grade PCa tissue samples were stained for *TAPIR-1* (A-D), *TAPIR-2* (E-H), PSA (as a positive control; I,J), without primary probes (K) and with a bacterial lacZ specific probe (as a negative control; L) by FastBlue dye (blue spots). Counterstaining was done using nuclear fast red (red). Positive *TAPIR-1* and *-2* staining was observed in PCa (PCa: A-B, E-F, respectively) and prostatic intraepithelial neoplasia (PIN: C, G) but not in connective tissue. BPH content showed no to weak staining (D, H). Inlays (A-D, E-F, and J) show areas (marked with arrows) at higher magnifications.

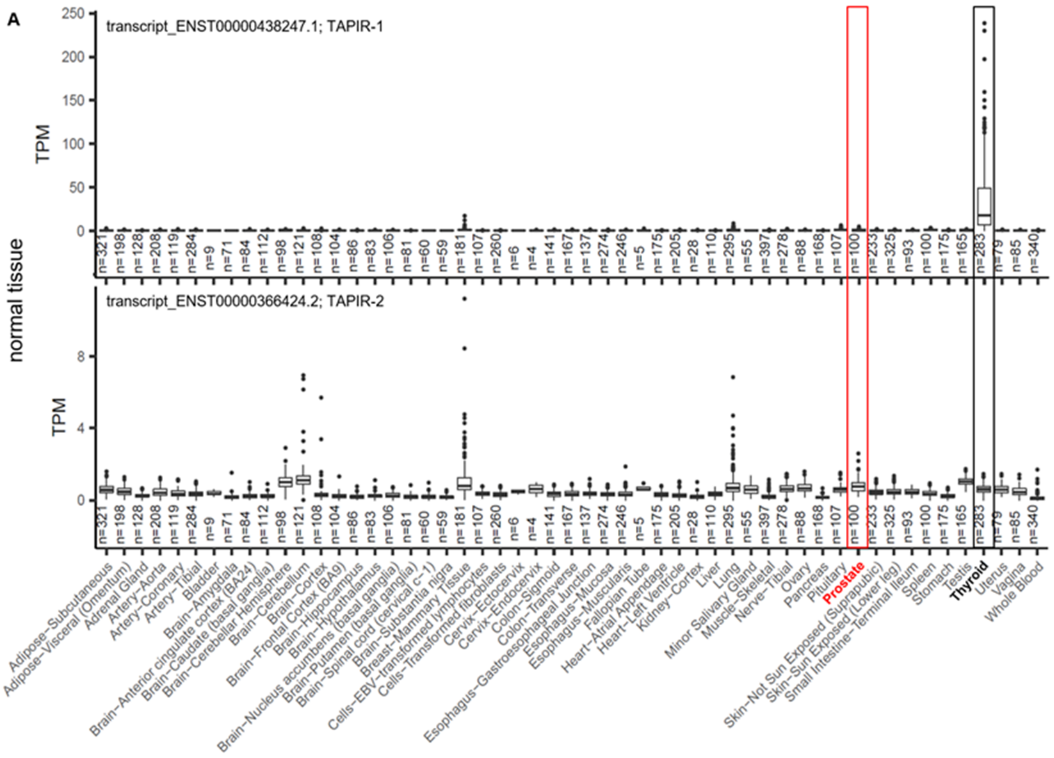

Figure S4. *TAPIR-1* and *-2* expression is low in normal tissue. (**A**). Gene expression distribution for *TAPIR-1* (ENST00000438247.1) and *TAPIR-2* (AC144450.2, ENST00000366424.2) in normal tissues was analyzed from public transcriptome sequencing gene data analyses from 53 normal tissues (8555 samples; 570 donors) of the Genotype Tissue Expression (GTEx) project. Expression values are shown as TPM (Transcripts Per Million) calculated from a gene model with all isoforms collapsed to single genes. No other normalization steps have been applied. Box plots are shown as median and 25th and 75th percentiles. Prostate (red) and thyroid (black) tissue are marked by red and black box, respectively.

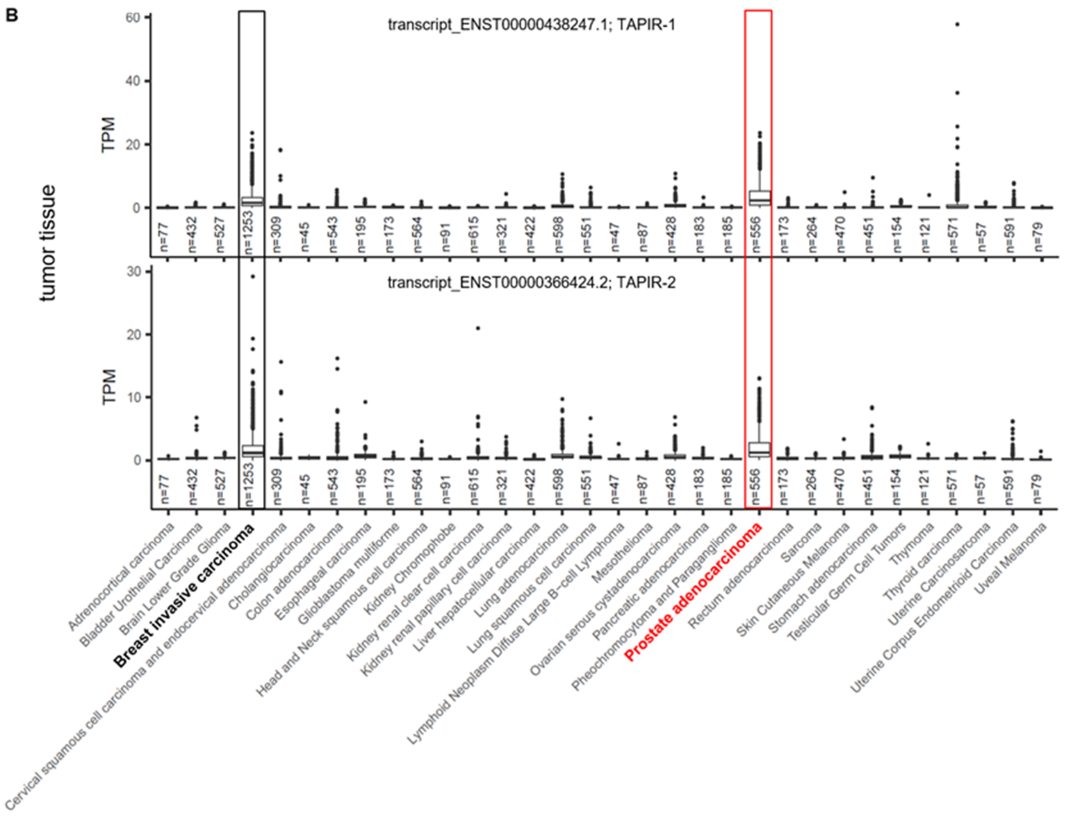

Figure S5. *TAPIR-1* and *-2* overexpression is restricted to tumor tissue. Gene expression distribution for *TAPIR-1* (ENST00000438247.1) and *TAPIR-2* (ENST00000366424.2) in cancer tissues were analyzed from RNA-seq expression data from 32 different tumor entities (11,133 samples) provided by the Cancer Genome Atlas (TCGA; https://cancergenome.nih.gov/). Expression values are shown in TPM calculated from gene model with isoforms collapsed to single genes. No other normalization steps have been applied. Box plots are shown as median and 25th and 75th percentiles. Prostate adenocarcinoma (red) and breast invasive carcinoma (black) specimens are marked by red and black box, respectively.

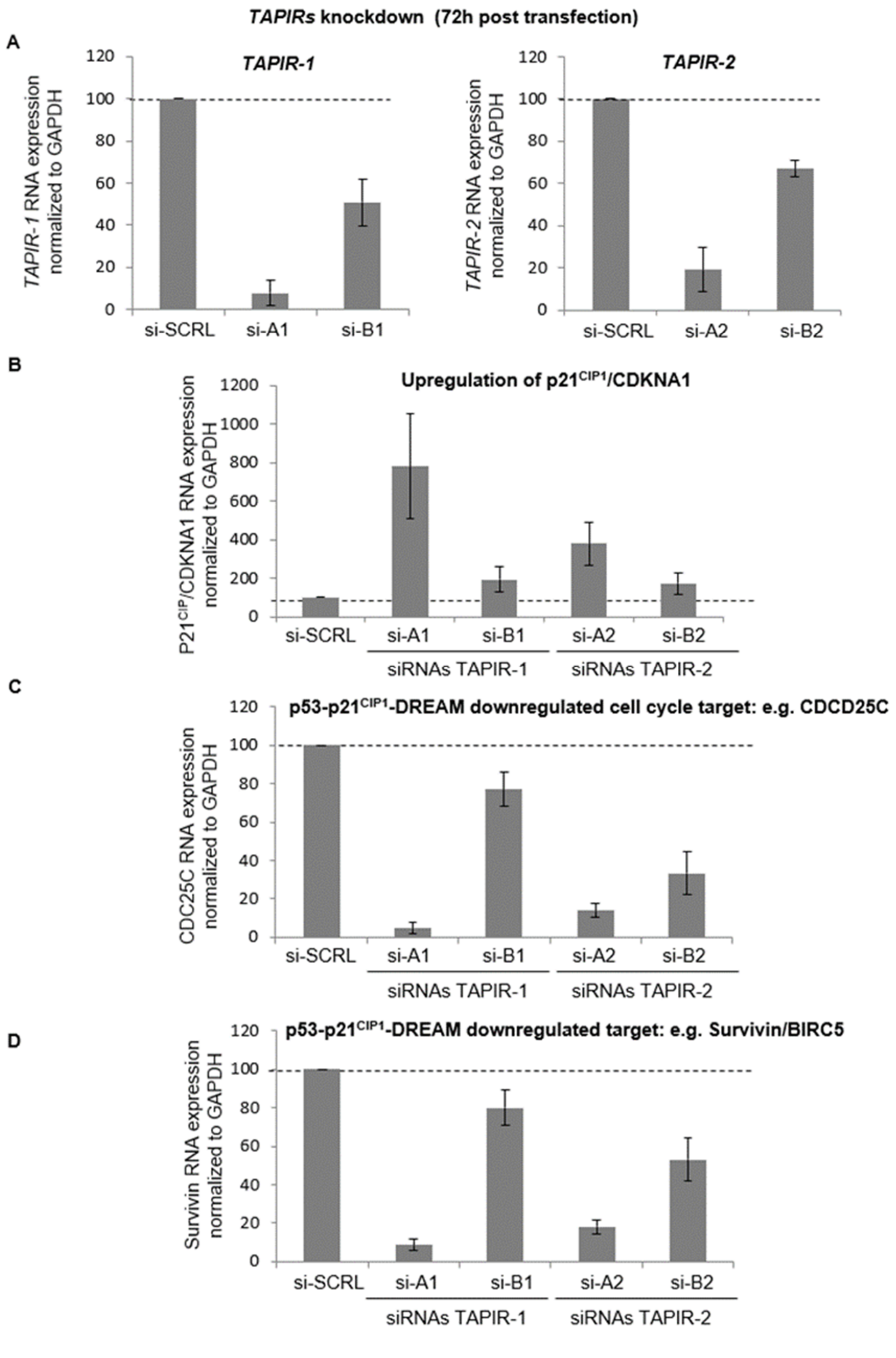

Figure S6. *TAPIRs* knockdown with 4 different *TAPIR*-directed siRNAs in LNCaP cells. Knockdown efficacy of two different *TAPIR-1* siRNAs (si-A1, si-B1) and two different *TAPIR-2* siRNAs (si-A2, si-B2) is shown 72h after transfection. RNA was isolated and reverse transcribed into cDNA. The expression levels of *TAPIR-1*, *TAPIR-2*, CDC25C, and Survivin/BIRC-5 were determined by qPCR (normalized to GAPDH). Data represent the mean±s.d. of at least n=3 biological replicates, measured in triplicate.

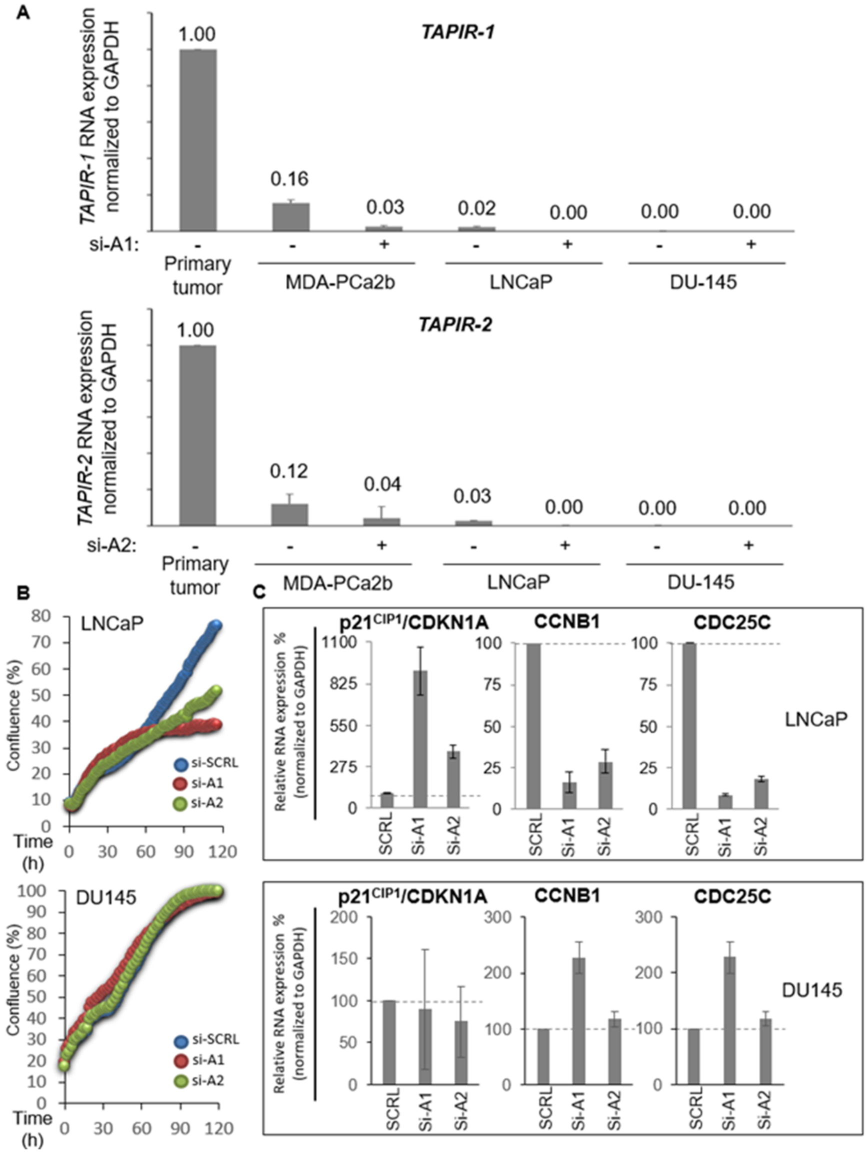

Figure S7. Comparison between primary tumors and cell lines and siRNA knockdown based phenotype of TAPIR lncRNAs positive and negative cells. (A) Expression pattern of TAPIR-1 and -2 and knockdown efficacy siRNAs (si-A1, si-B1) is shown 72h after transfection. The expression levels were normalized and compared to those of primary PCa tumor tissue. Experimentally, RNA was isolated, reverse transcribed into cDNA and the expression of *TAPIR-1*, and -2were determined by qPCR (normalized to GAPDH) an compared to those of primary PCa tumor tissue. Data represent the mean±s.d. of at least n=3 biological replicates, measured in triplicate. (B) IncuCyte live cell analysis. LNCaP- and DU145 cells were siRNA-transfected and seeded into ImageLock plates. Cell were grown for 6 days and monitored by time-lapse microscopy. The results of the IncuCyte experiments was quantified by cell confluency and shown as blue, red, and green filled circles indicating SCRL, si-A1, and si-A2, respectively. (C) Comparison of knockdown phenotype between LNCaP and DU145 cells. RNA was isolated LNCaP- and DU145 cells, reverse transcribed into cDNA. The expression levels of p21^{CIP1}/CDKNA1, CDC25C, and CCNB1 were determined by qPCR (normalized to GAPDH). Data represent the mean±s.d. of n=3 biological replicates, measured in triplicate. Note, that DU145 cells does not express significant levels of TAPIR-1 and -2. Transfection of DU145 cells with TAPIRs specific siRNAs does not alter their proliferation and does not lead to an upregulation of p21^{CIP1}/CDKN1A and a downregulation of cell cycle key regulators like CCNB1 and CDC25C.

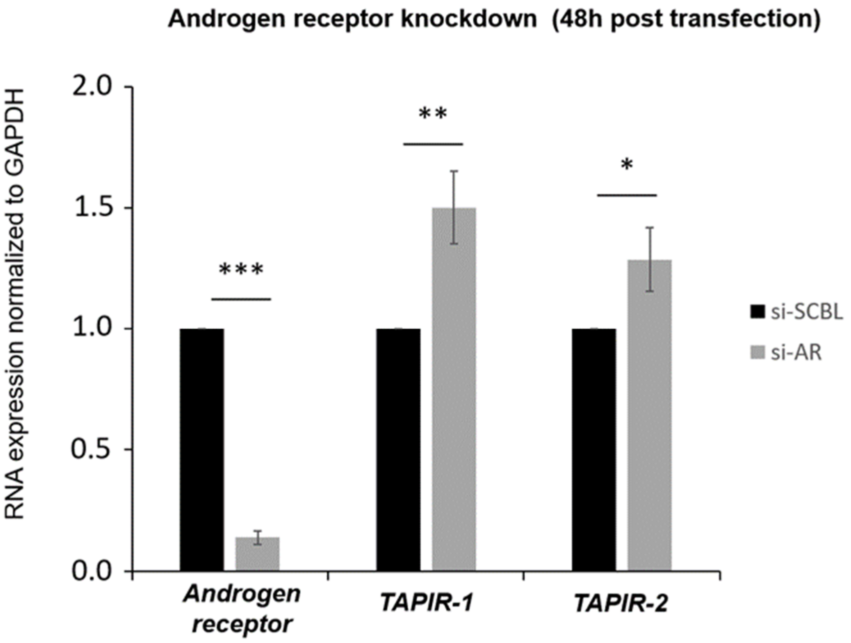

Figure S8. Expression pattern of *TAPIR-1* and *-2* after androgen receptor knockdown. (**A**) LNCaP cells were cultured and treated with siRNA to knockdown androgen receptor (AR) and siRNA control (SCBL). Knockdown efficacy of siRNAs is shown 48h after transfection. RNA was isolated from cells and reverse transcribed into cDNA. The expression levels of AR, *TAPIR-1* and *-2* were determined by qPCR and normalized to *GAPDH*. Data represent the mean±s.d. of n=4 biological replicates. Significance $p \leq 0.001$ (***), $p \leq 0.01$ (***), $p \leq 0.05$ (*); student-t test.

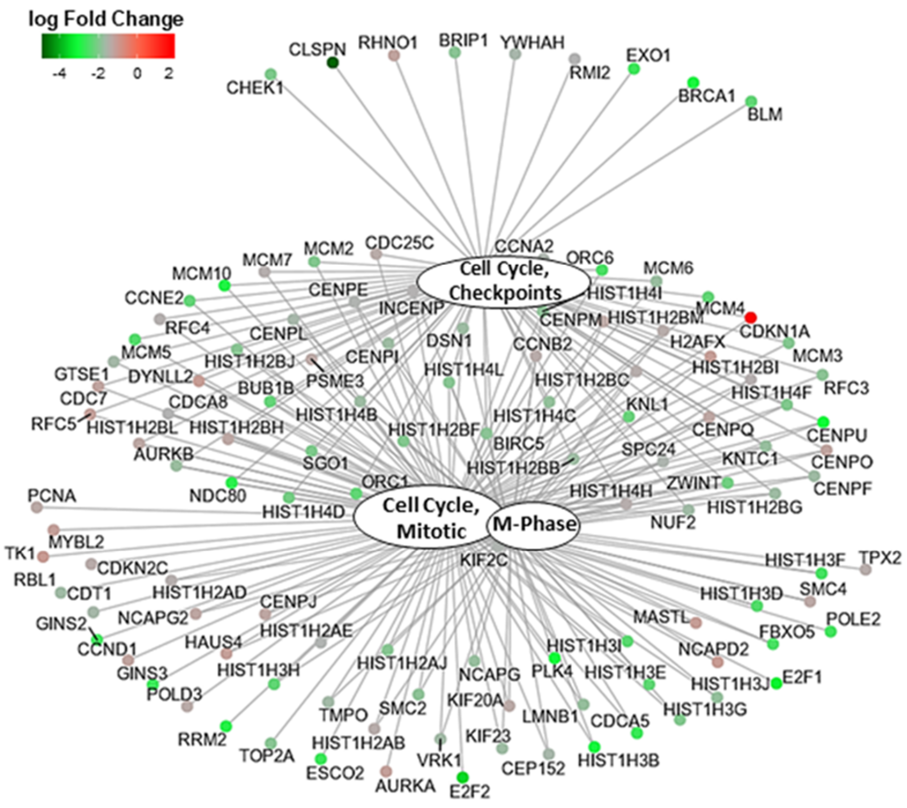

Figure S9. Enlarge graph of the ReactomePA pathway analysis of Figure 4A

A Microarray pathway analysis; Cell cycle, Mitosis and Checkpoint genes 24h after *TAPIR-1* knockdown

Gene	logFC	pV	Gene	logFC	pV	Gene	logFC	pV
CLSPN	-3.8	***	CCND1	-1.8	***	GTSE1	-1.3	***
HIST1H1B	-3.5	***	KNTC1	-1.8	***	HIST1H2AM	-1.3	**
RRM2	-3.1	***	CCNB2	-1.8	***	RFC5	-1.3	***
BRCA1	-2.8	***	CDCA2	-1.8	***	HIST1H2AD	-1.3	**
ESCO2	-2.8	***	CENPI	-1.8	***	CENPL	-1.3	**
PLK4	-2.8	***	CHEK1	-1.7	***	INCENP	-1.3	**
ORC6	-2.7	***	RFC3	-1.7	***	CENPE	-1.3	**
HIST1H3B	-2.7	***	NCAPD2	-1.7	***	SPDL1	-1.3	***
CENPU	-2.7	***	CENPA	-1.7	**	RBL1	-1.2	**
HIST1H2AI	-2.7	***	TOP2A	-1.7	***	HIST1H2BI	-1.2	**
MCM10	-2.6	***	AURKB	-1.7	***	HIST1H4C	-1.2	**
GINS2	-2.6	***	CDCA5	-1.7	**	HIST1H1C	-1.2	**
GINS3	-2.5	***	HIST1H4L	-1.7	***	CENPM	-1.2	***
E2F1	-2.5	***	HIST1H4B	-1.6	***	CDCA7L	-1.2	**
ORC1	-2.5	***	NCAPG	-1.6	***	CDKN2C	-1.2	**
BLM	-2.4	***	H2AFV	-1.6	***	HIST1H2BM	-1.2	**
NDC80	-2.4	**	SMC2	-1.6	**	HIST1H2AG	-1.2	**
DSN1	-2.4	***	MCM2	-1.6	***	HIST1H2BL	-1.2	**
CDCA7	-2.4	***	RFC4	-1.6	***	HIST1H2BH	-1.2	**
HIST1H3F	-2.4	***	NUF2	-1.6	***	SPC24	-1.2	**
EXO1	-2.3	***	TYMS	-1.6	***	TK1	-1.1	**
HIST1H3D	-2.3	***	HIST1H4H	-1.6	***	CENPJ	-1.1	**
E2F2	-2.2	**	CDK2	-1.6	**	HIST1H2BC	-1.1	**
KNL1	-2.2	***	CENPQ	-1.6	***	KIF2C	-1.1	***
NCAPG2	-2.2	***	CCNE2	-1.6	***	HIST1H2AB	-1.1	**
HIST1H3J	-2.2	***	PRIM2	-1.6	**	TPX2	-1.1	**
MCM5	-2.1	***	KIF23	-1.6	***	KIF20A	-1.1	**
HIST1H3I	-2.1	***	MCM6	-1.5	***	PLK1	-1.1	**
HIST1H3H	-2.1	***	HIST1H1E	-1.5	**	AURKA	-1.1	**
HIST1H3E	-2.1	***	HIST1H2BJ	-1.5	***	SMC1A	-1.0	**
SKP2	-2.1	***	CDT1	-1.5	***	BRMS1	-0.9	**
BIRC5	-2.1	***	HIST1H2AE	-1.5	***	MASTL	-0.9	**
POLE2	-2.1	***	MCM3	-1.4	***	DYNLL2	-0.8	**
MCM4	-2.0	***	CDCA8	-1.4	***	E2F5	1.2	**
HIST1H4F	-2.0	***	VRK1	-1.4	**			
HIST1H4D	-2.0	***	HIST1H2BB	-1.4	**			
ZWINT	-2.0	***	SMC4	-1.4	***			
HIST1H2AJ	-2.0	***	HIST1H2BG	-1.4	***			
HIST1H3G	-1.9	***	CCNA2	-1.4	***			
BUB1B	-1.9	***	PCNA	-1.4	***			
SGO1	-1.9	**	CENPF	-1.4	**			
HIST1H2AK	-1.9	***	RMI2	-1.3	***			
HIST1H4I	-1.9	***	CENPO	-1.3	***			
H2AFX	-1.9	***	MCM7	-1.3	***			

B Microarray KEGG pathway analysis

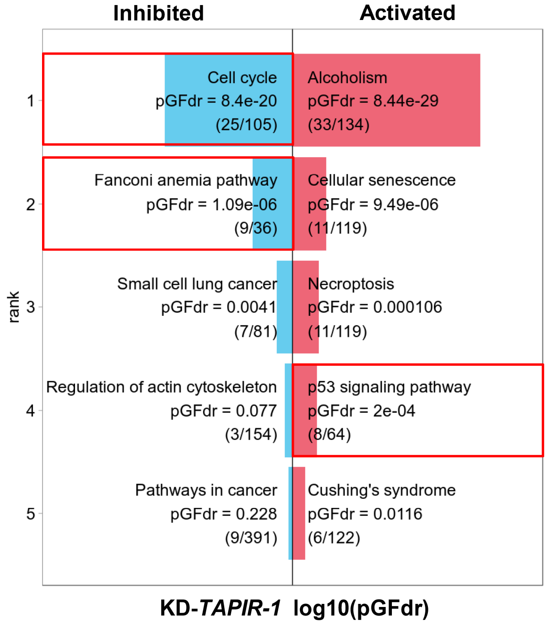

C Microarray heatmap

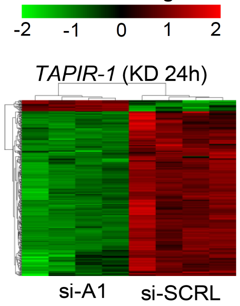

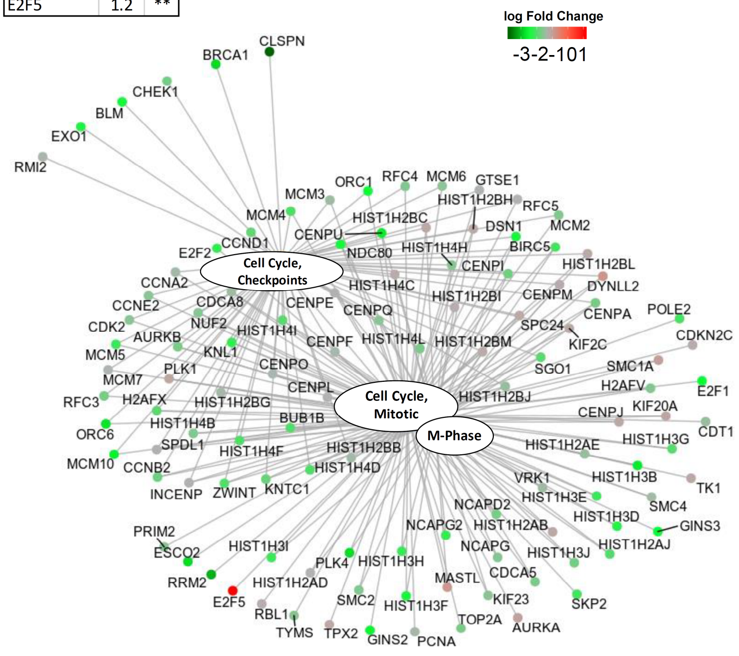

Figure S10. Increase in p53 levels and downregulation of cell cycle - and DNA repair control genes following *TAPIR-1* knockdown. (A) Differential mRNA expression following *TAPIR-1* knockdown. A microarray analysis was performed 24h after *TAPIR-1* and *-2* knockdowns. Data were analyzed and visualized using ReactomePA tool software to identify regulated target genes and cellular processes. An enlarged version of the graph is provided as supplemental Figure 11**.** ReactomePA is a pathway database tool used for the visualization, interpretation and analysis of regulated pathways. A table shows all cell cycle, mitosis and checkpoint regulated

genes 24 hours after *TAPIR-1* knockdown. Notably, this table includes 122 of the in total 317 significantly regulated genes. Adjusted p-values are given as p≤0.01 (**), p≤0.001 (***). Regulatory interactions are visualized by the function cnetplot() of the ReactomePA package. Downregulated genes are shown in green, upregulated genes in red. Shown are the identified regulated genes of the four most regulated cellular processes: cell cycle, mitotic, m-phase, and cell cycle checkpoints. Interactions of molecules are indicated by black lines. (B) Heatmap of differentially up- and down-regulated *TAPIR-1* target genes 24h post siRNA transfection determined by microarray analysis (performed in quadruplicate). Significant upregulated genes are shown in red, downregulated genes in green. Note that ~95% of the targets are downregulated. (C) Microarray signaling pathway impact analysis (SPIA). Transcriptome wide RNA expression patterns of siRNA treated LNCaP cells (each experiment was performed in quadruplicate) were measured by custom made microarray 24h after transfection. The biological pathways extraction was done by the use of Kyoto Encyclopedia of Genes and Genomes (KEGG) PATHWAY database. Downregulation of cell cycle and Fanconi anemia pathway genes (left red boxes) and upregulation of p53 signaling pathway (right red box) is shown. The false discovery rate-adjusted global probability (pGFdr) is given.

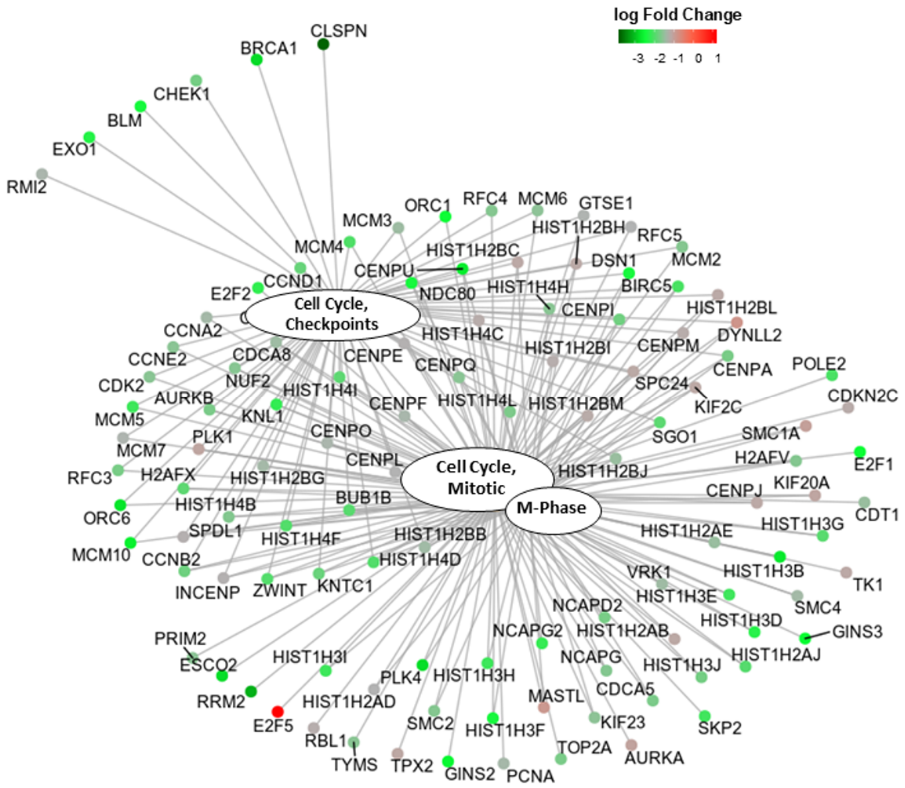

Figure S11. Enlarge graph of the ReactomePA pathway analysis of supplemental Figure 9

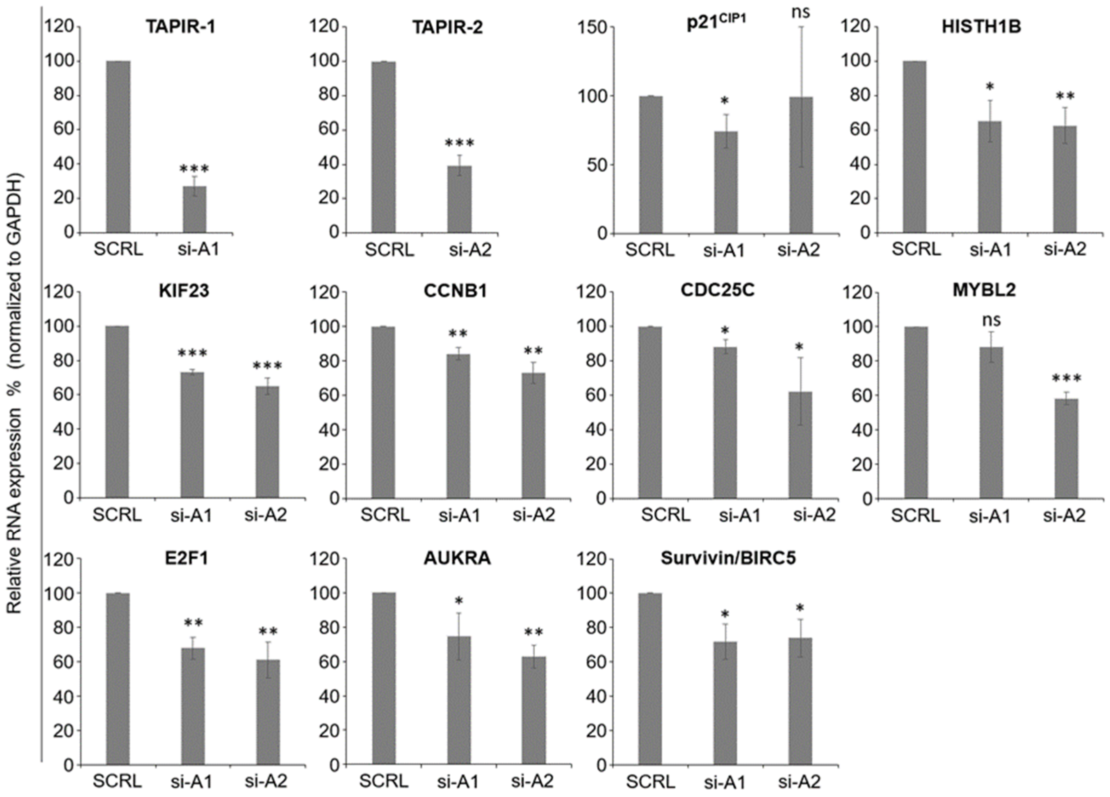

Figure S12. Validation of cell cycle regulating target genes after *TAPIR-1* and *-2* knockdown by qPCR. Total RNA was isolated from MDA-PCa2b cells 72h post siRNA transfection and reverse transcribed into cDNA. qPCR was performed to quantify *TAPIR-1*, *TAPIR-2*, p21^{CIP1}/CDKN1A, *CCNB1*, *KIF23*, *MYBL2*, *CDC25C*, *AURKA*, *HISTH1B*, *E2F1*, Survivin/*BIRC5*. The ΔCt values were determined by subtracting Ct value of housekeeper Ct value (GAPDH). Data are shown as means±s.d.; Significance p≤0.05 (*), p≤0.01 (**), p≤0.001 (***), ns = not significant; two-sided student-t test.

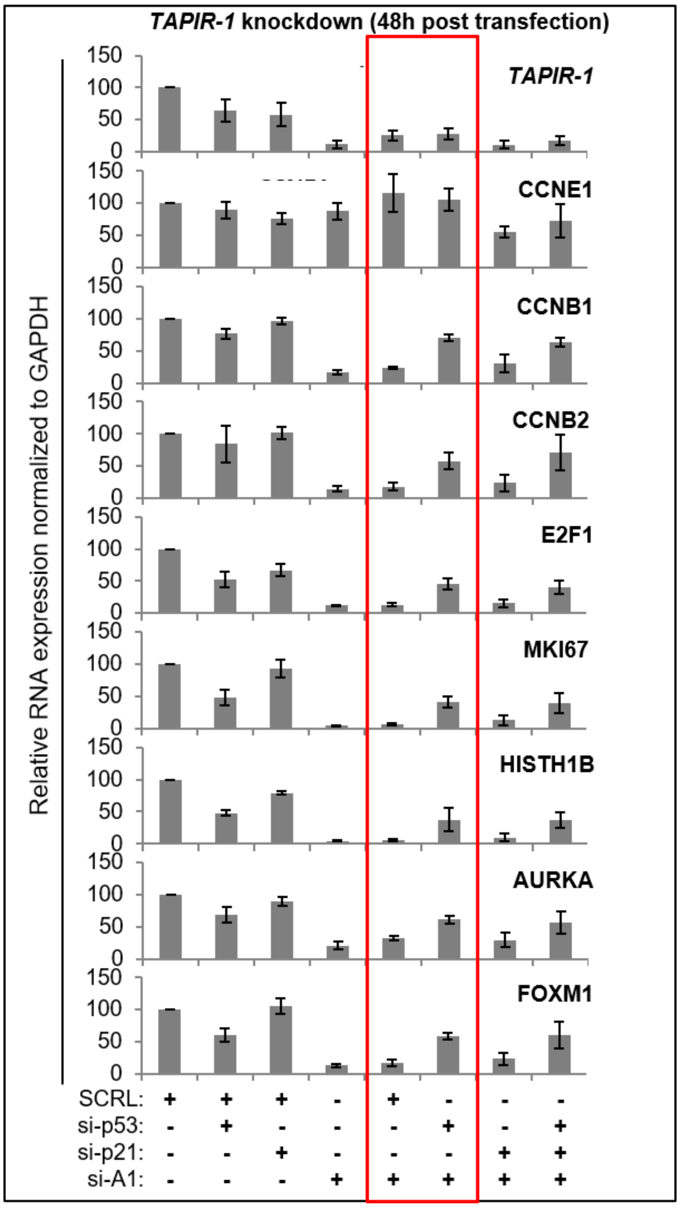

Figure S13. Gene expression alterations after knockdown of p53 or *TAPIR-1* 48h post siRNA transfection. LNCaP cells were transfected with siRNAs against *TAPIR-1*, p53/TP53, p21^{CIP1}/CDKN1A, scrambled control (and combinations) and cultured for 48h. Results of qPCR analysis of *TAPIR-1*, CCNE1, CCNB1, CCNB2, E2F1, MKI67, HISTH1B, AURKA and FOXM1 are shown. The ΔCt values were determined by subtracting Ct value of housekeeper Ct value (GAPDH). Each qPCR reaction was performed in triplicate at least with three independent biological replicates. Data are shown as means±s.d..

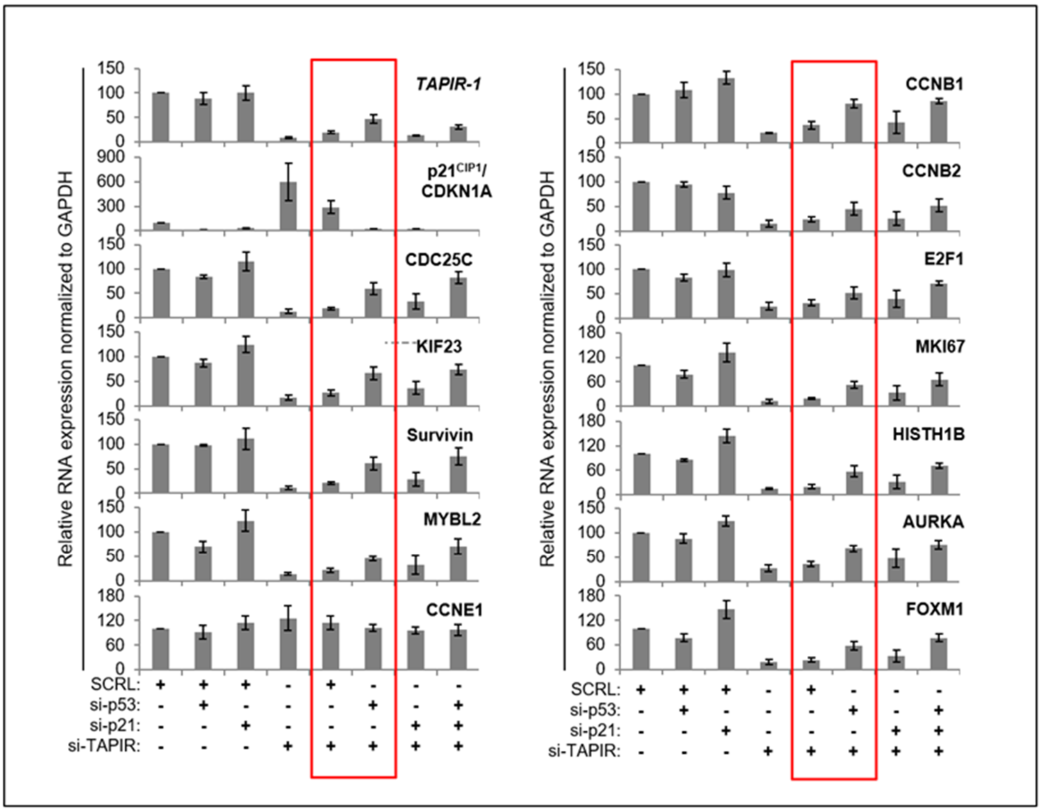

Figure S14. Gene expression alterations after knockdown of p53 or *TAPIR-1* 72h post siRNA transfection. LNCaP cells were transfected siRNA against *TAPIR-1*, p53/TP53, p21^{CIP1}/CDKN1A, scramble control (and combinations) and cultured for 72h. Results of qPCR analysis of *TAPIR-1*, p21^{CIP1}/CDKN1A, CDC25C, KIF23, Survivin/BIRC-5, MYBL2, CCNE1, CCNB1, CCNB2, E2F1, MKI67, HISTH1B, AURKA and FOXM1 are shown. The ΔCt values were determined by subtracting Ct value of housekeeper Ct value (GAPDH). Each qPCR reaction was performed in triplicate at least with three independent biological replicates. Data are shown as means±s.d.

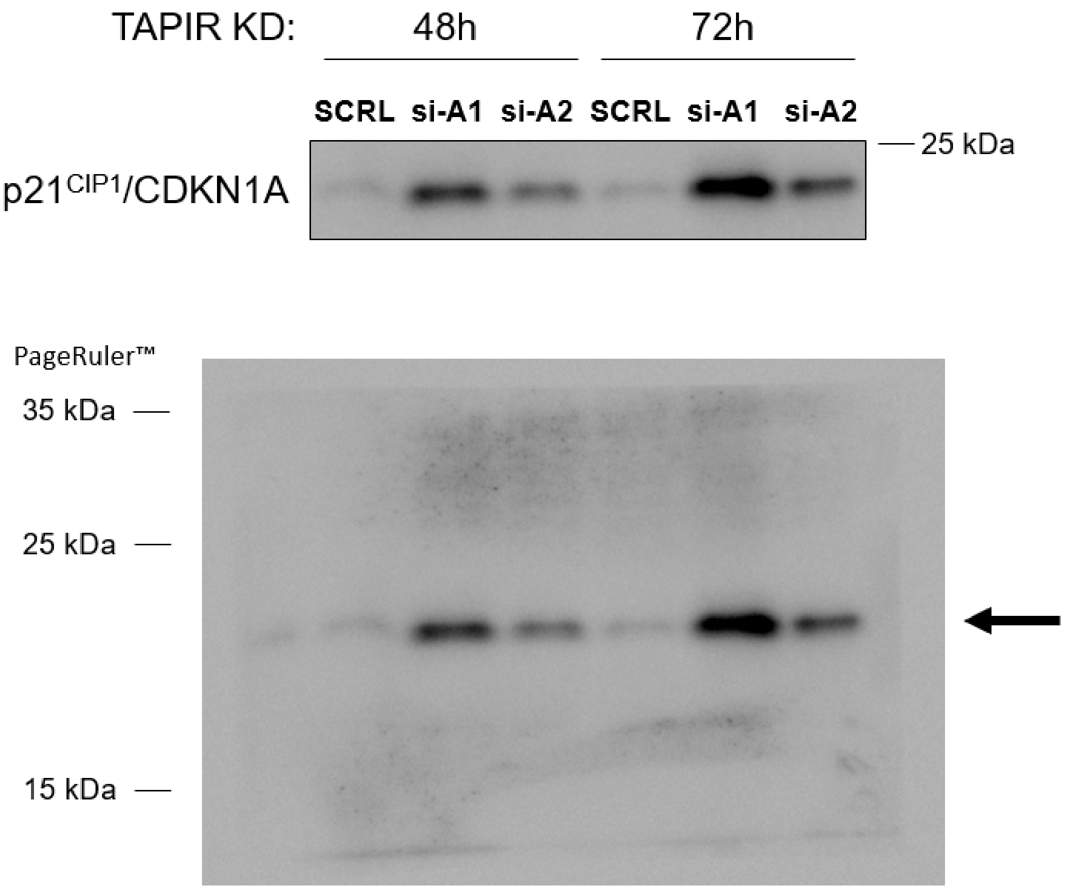
TAPIR KD:
48h
72h
SCRL si-A1 si-A2 SCRL si-A1 si-A2
p21CIP1/CDKN1A
25 kDa
PageRuler™
35 kDa
25 kDa
15 kDa

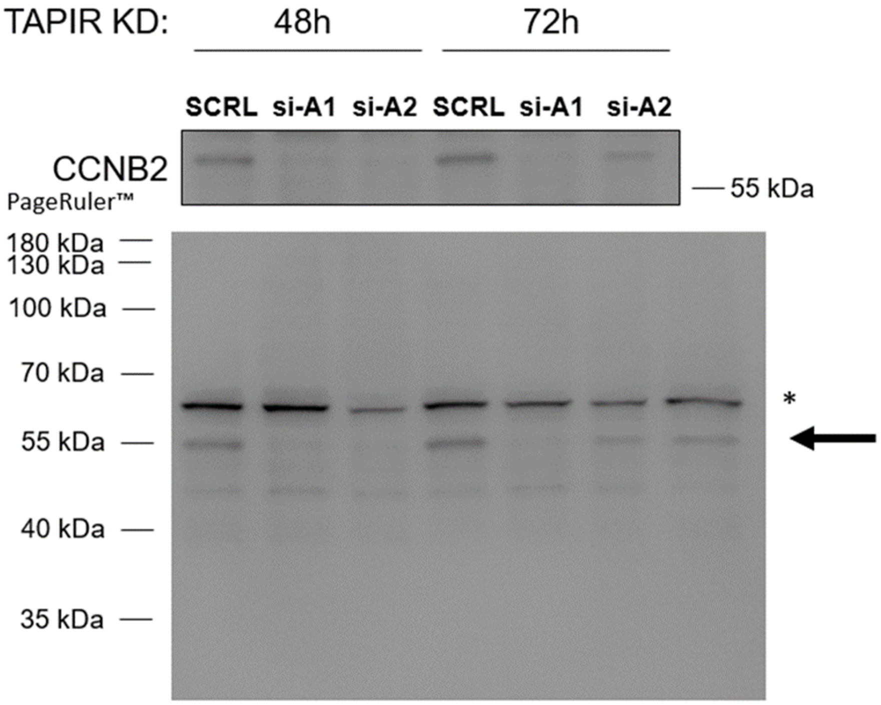
TAPIR KD:
48h
72h
SCRL si-A1 si-A2 SCRL si-A1 si-A2
CCNB2
PageRuler™
55 kDa
180 kDa
130 kDa
100 kDa
70 kDa
55 kDa
40 kDa
35 kDa
*

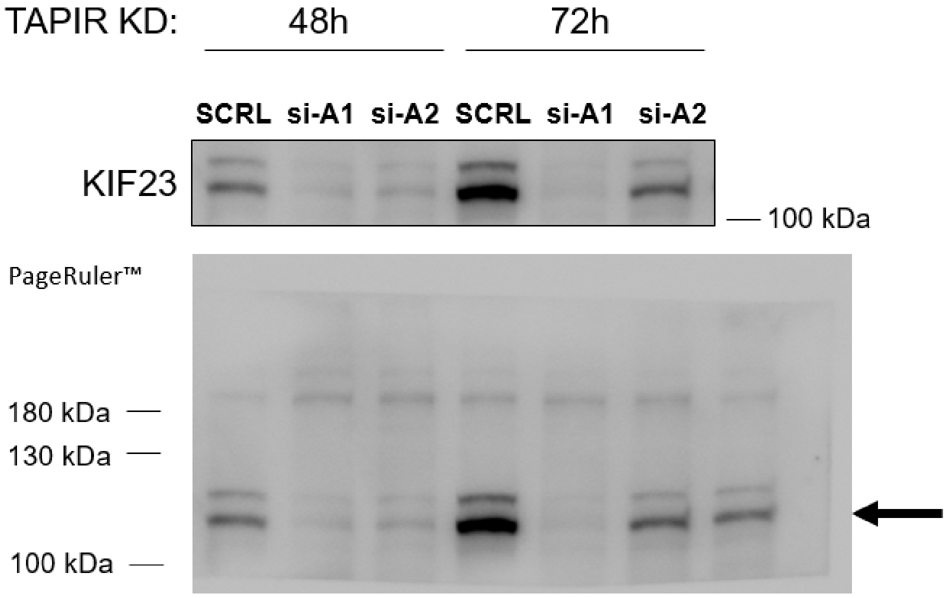
TAPIR KD:
48h
72h
SCRL si-A1 si-A2 SCRL si-A1 si-A2
KIF23
100 kDa
PageRuler™
180 kDa
130 kDa
100 kDa

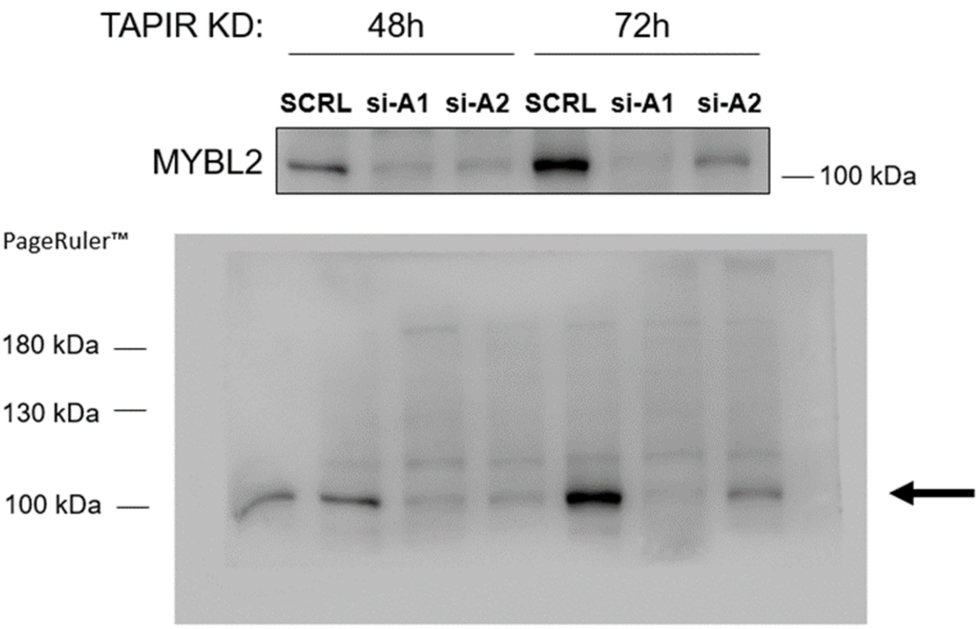
TAPIR KD:
48h
72h
SCRL
si-A1
si-A2
SCRL
si-A1
si-A2
MYBL2
100 kDa
PageRuler™
180 kDa
130 kDa
100 kDa

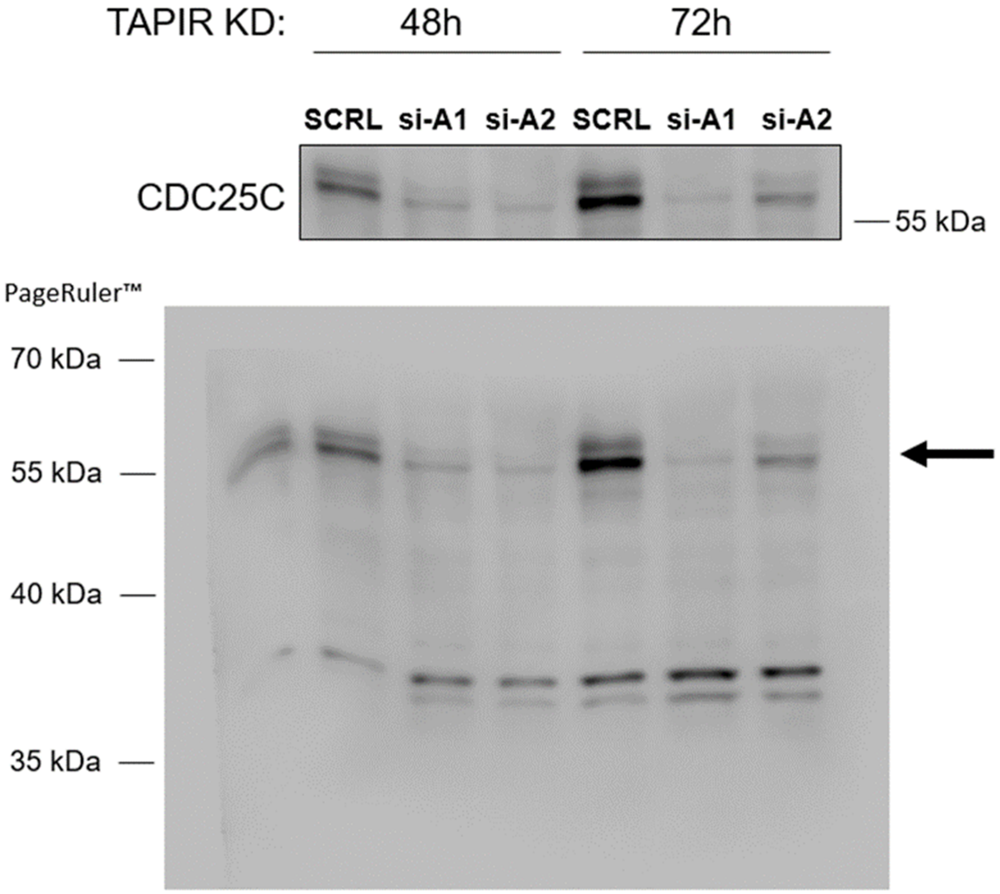
TAPIR KD:
48h
72h
SCRL
si-A1
si-A2
SCRL
si-A1
si-A2
CDC25C
55 kDa
PageRuler™
70 kDa
55 kDa
40 kDa
35 kDa

CDC25C

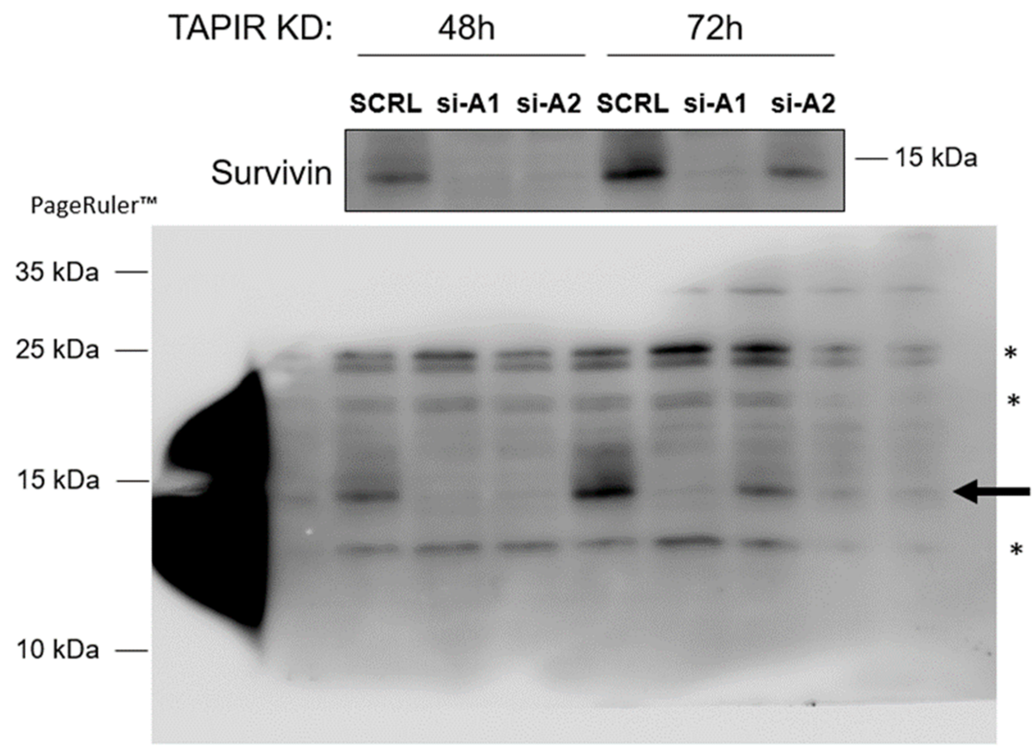
TAPIR KD:
48h
72h
SCRL
si-A1
si-A2
SCRL
si-A1
si-A2
Survivin
15 kDa
PageRuler™
35 kDa
25 kDa
15 kDa
10 kDa
*
*
*

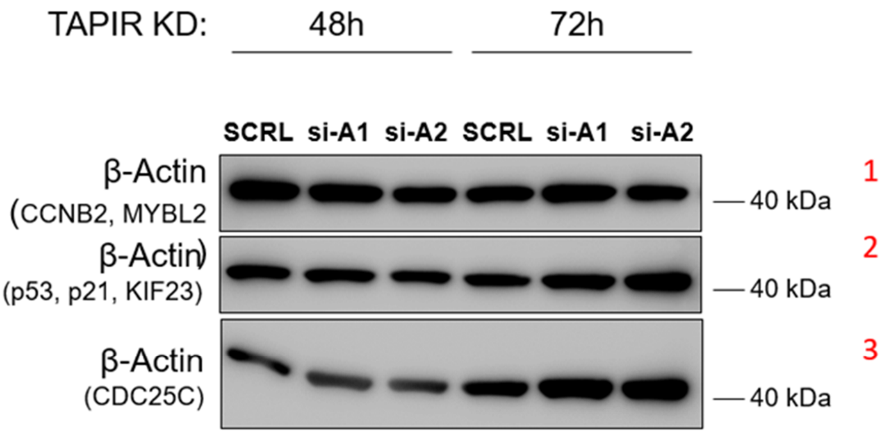
TAPIR KD:
48h
72h
SCRL
si-A1
si-A2
SCRL
si-A1
si-A2
β-Actin
(CCNB2, MYBL2
β-Actin)
(p53, p21, KIF23)
β-Actin
(CDC25C)
40 kDa
40 kDa
40 kDa
1
2
3

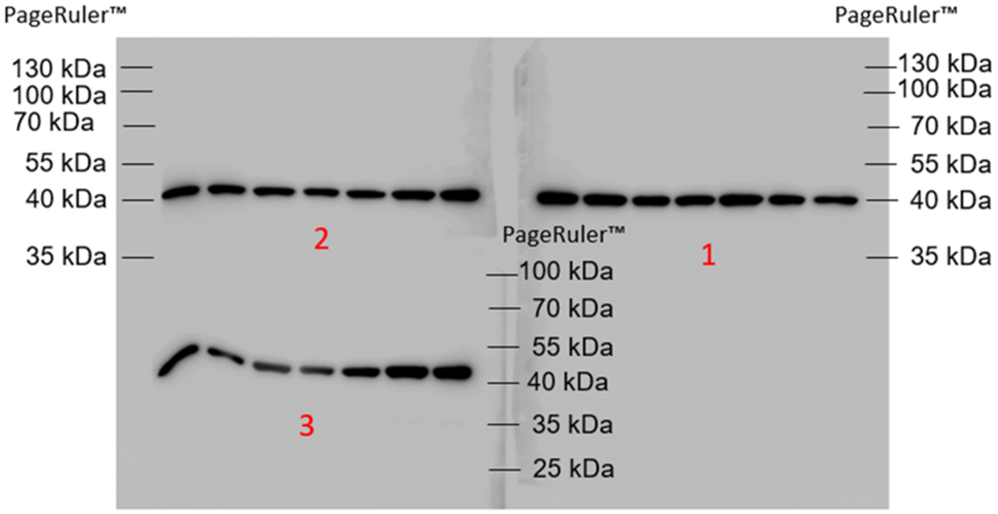
PageRuler™
130 kDa
100 kDa
70 kDa
55 kDa
40 kDa
35 kDa
2
3
PageRuler™
100 kDa
70 kDa
55 kDa
40 kDa
35 kDa
25 kDa
1
PageRuler™
130 kDa
100 kDa
70 kDa
55 kDa
40 kDa
35 kDa

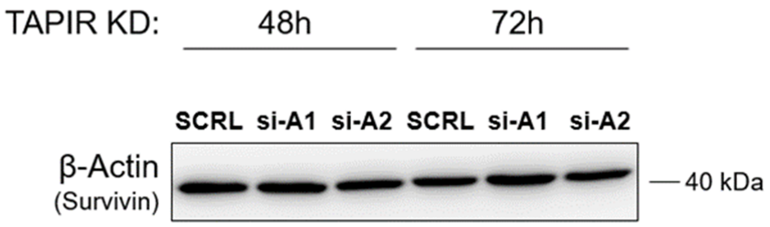

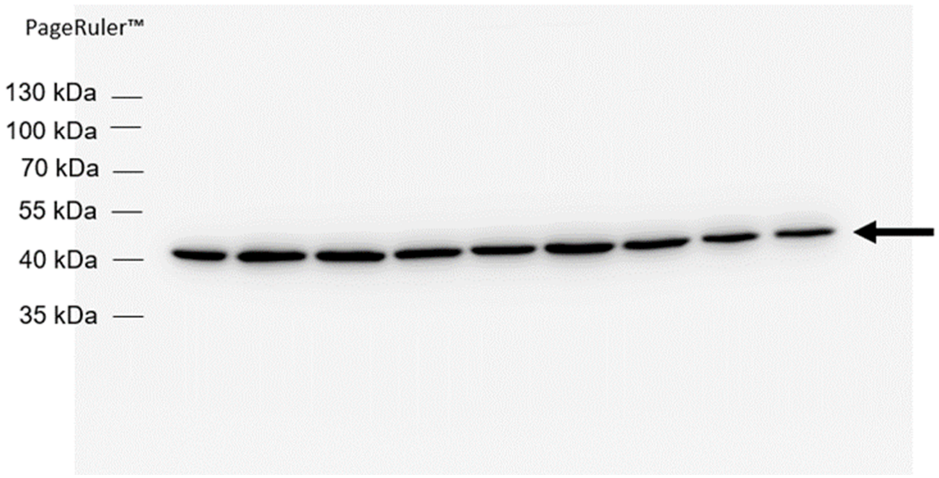

Uncropped western blot images of *TAPIR-1* target gene protein expression is shown. Cropped images (lanes) focusing on the specific protein are presented in **Figure 5c.**

Figure S15. Uncropped western blot images

Uncropped western blot images of *TAPIR-1* and *-2* target gene protein expression are shown. Cropped images (lanes) focusing on the specific protein are presented in **Figure 5a.**

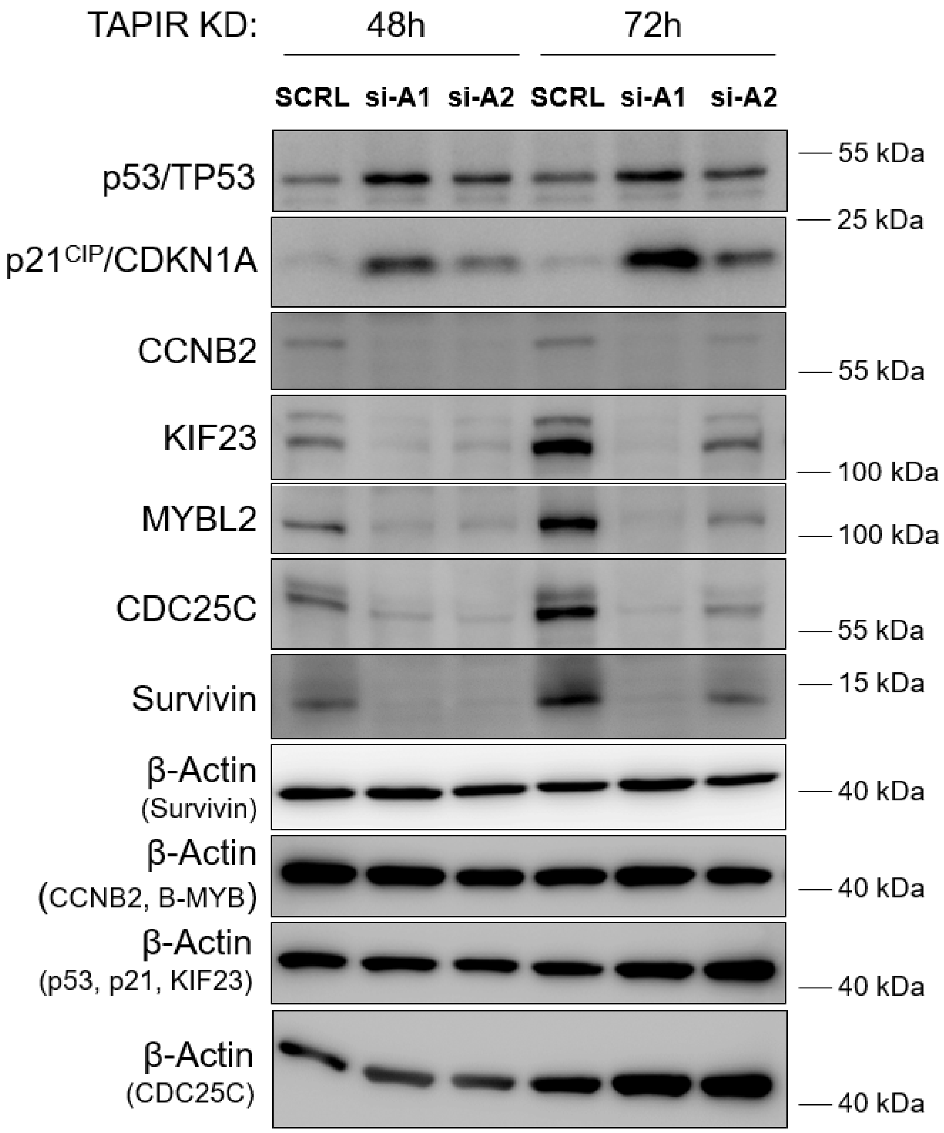

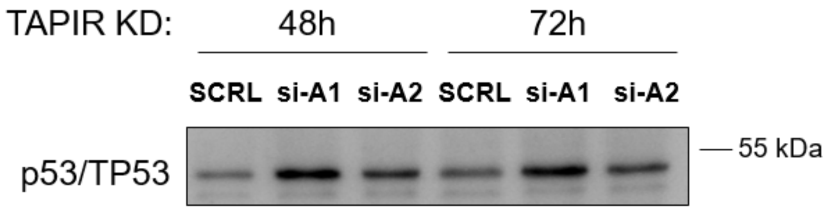
TAPIR KD:
48h
72h
SCRL si-A1 si-A2 SCRL si-A1 si-A2
p53/TP53
55 kDa

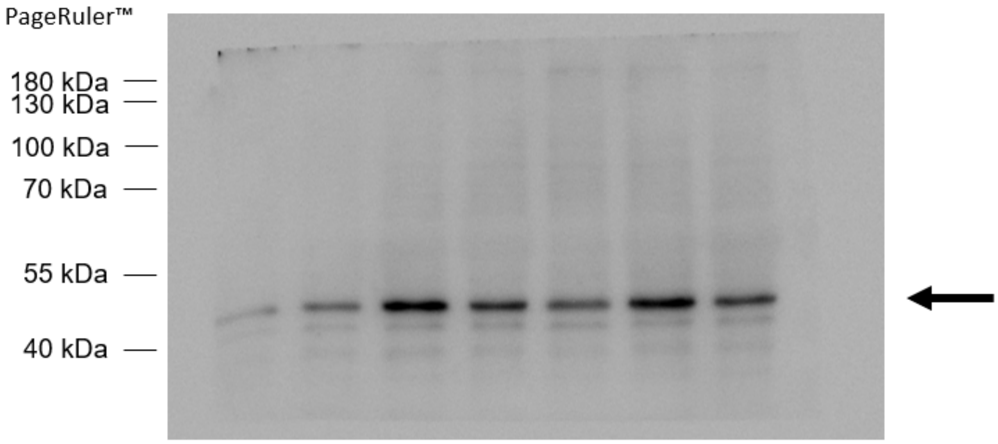
PageRuler™
180 kDa
130 kDa
100 kDa
70 kDa
55 kDa
40 kDa

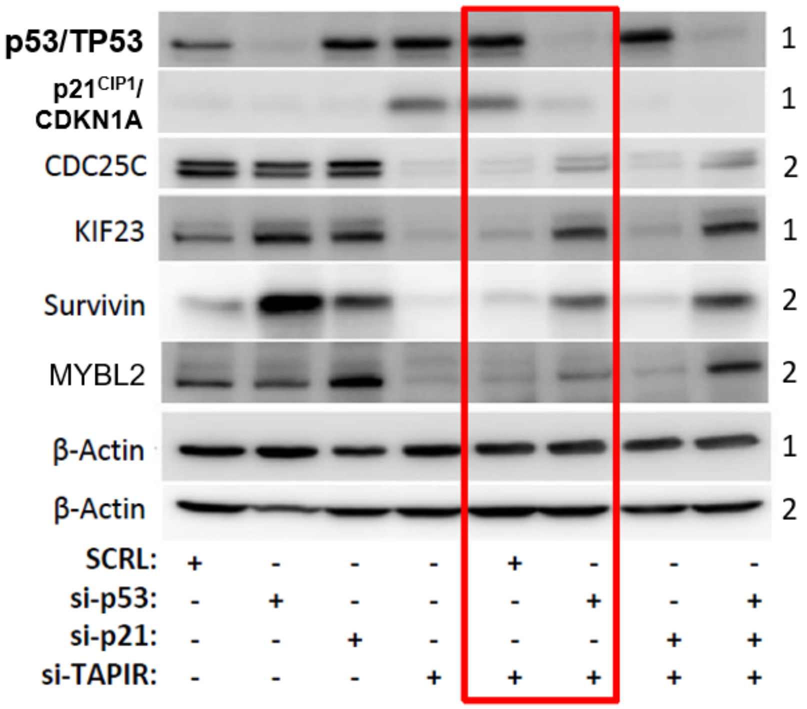
p53/TP53
1
p21CIP1/
CDKN1A
1
CDC25C
2
KIF23
1
Survivin
2
MYBL2
2
β-Actin
1
β-Actin
2
SCRL: + - - - + - - -
si-p53: - + - - - + - +
si-p21: - - + - - - + +
si-TAPIR: - - - + + + + +

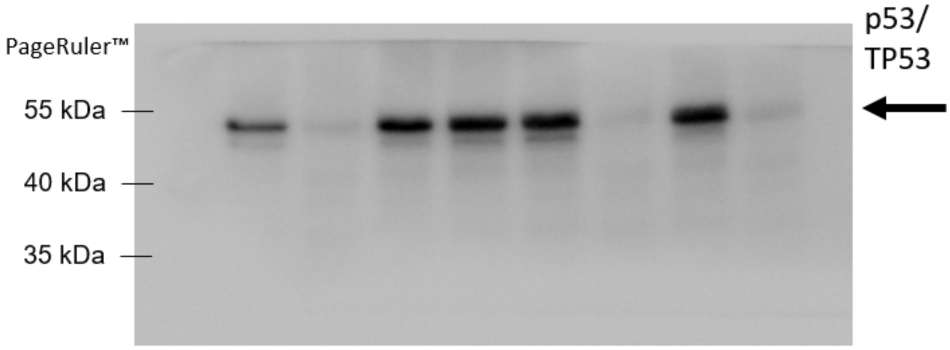
PageRuler™
55 kDa
40 kDa
35 kDa
p53/
TP53

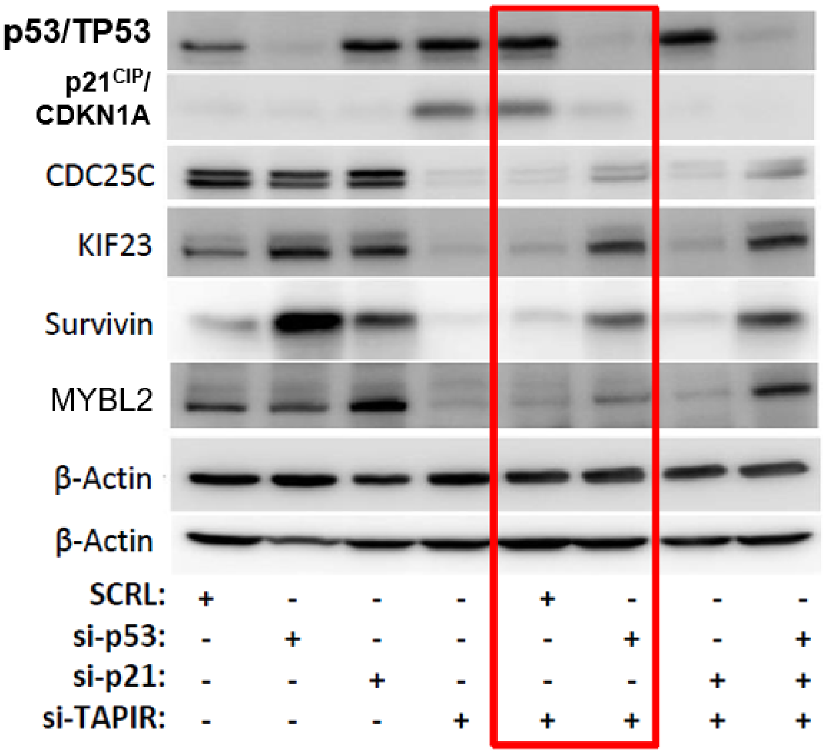
p53/TP53
p21CIP/ CDKN1A
CDC25C
KIF23
Survivin
MYBL2
β-Actin
β-Actin
SCRL: + - - - + - - -
si-p53: - + - - - + - +
si-p21: - - + - - - + +
si-TAPIR: - - - + + + + +

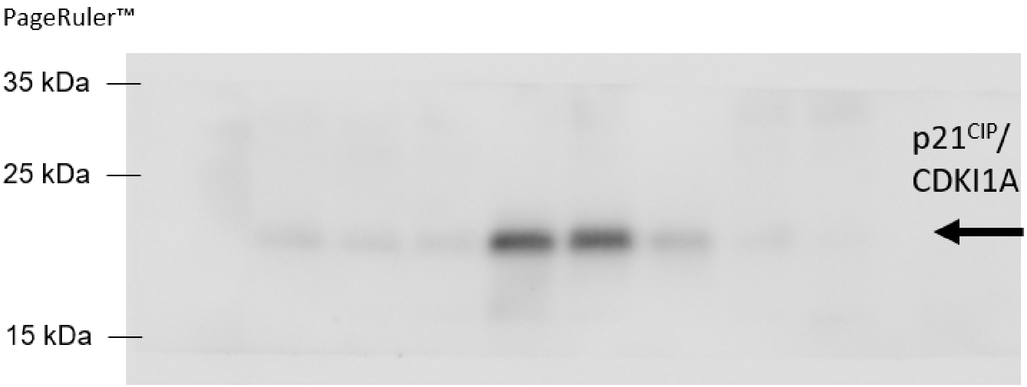
PageRuler™
35 kDa
25 kDa
15 kDa
p21CIP/ CDKI1A

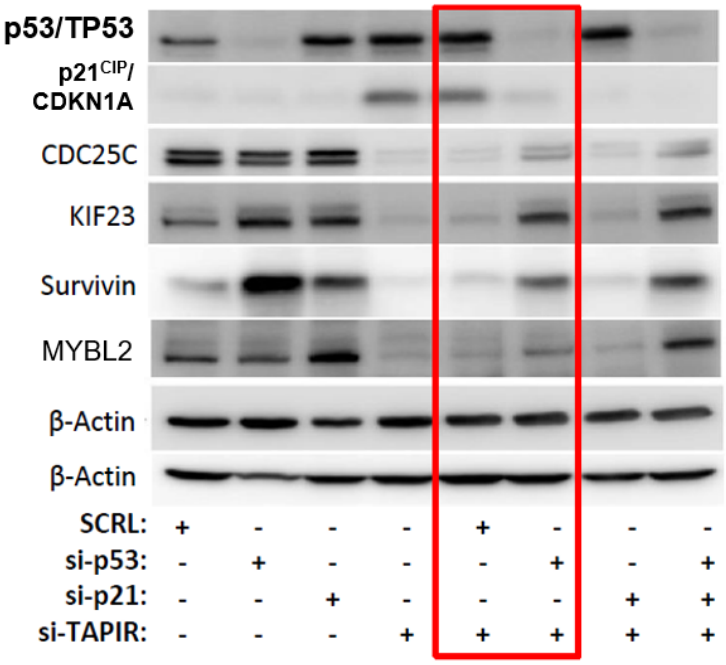
p53/TP53
p21CIP/
CDKN1A
CDC25C
KIF23
Survivin
MYBL2
β-Actin
β-Actin
SCRL: + - - - + - - -
si-p53: - + - - - + - +
si-p21: - - + - - - + +
si-TAPIR: - - - + + + + +

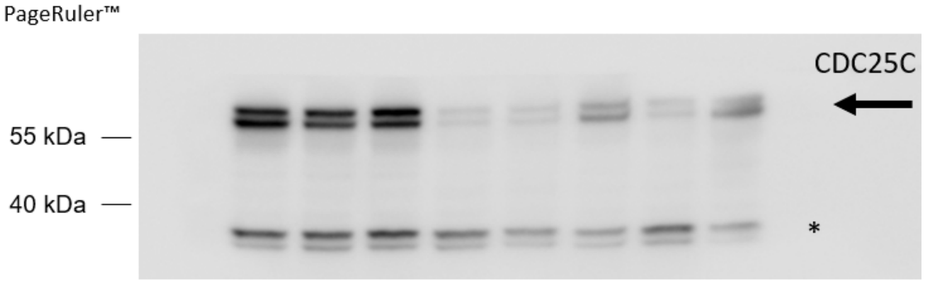
PageRuler™
55 kDa
40 kDa
CDC25C
*

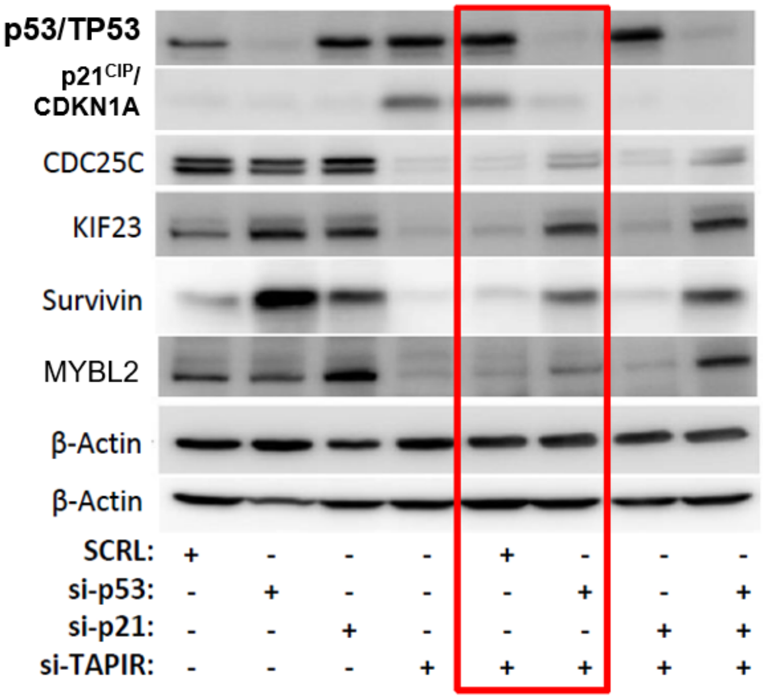
p53/TP53
p21CIP/
CDKN1A
CDC25C
KIF23
Survivin
MYBL2
β-Actin
β-Actin
SCRL: + - - - + - - -
si-p53: - + - - - + - +
si-p21: - - + - - - + +
si-TAPIR: - - - + + + + +

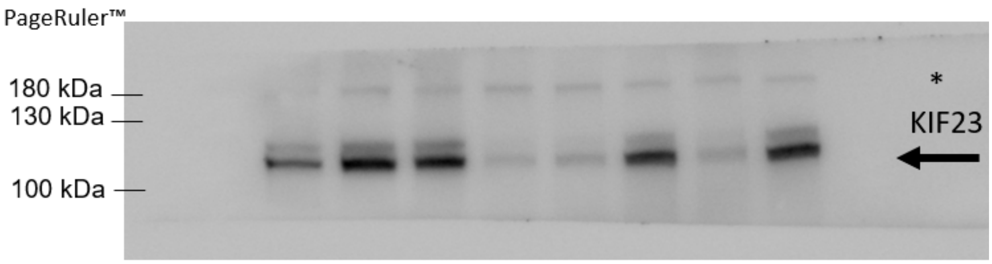
PageRuler™
180 kDa
130 kDa
100 kDa
*
KIF23

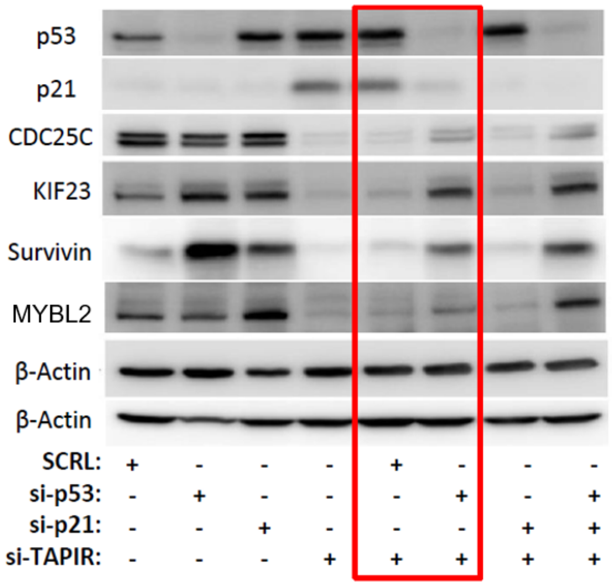
p53
p21
CDC25C
KIF23
Survivin
MYBL2
β-Actin
β-Actin
SCRL: + - - - + - - -
si-p53: - + - - - + - +
si-p21: - - + - - - + +
si-TAPIR: - - - + + + + +

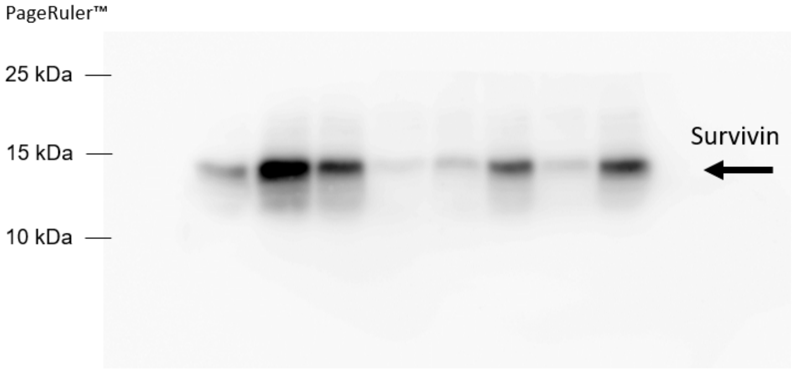
PageRuler™
25 kDa
15 kDa
10 kDa
Survivin

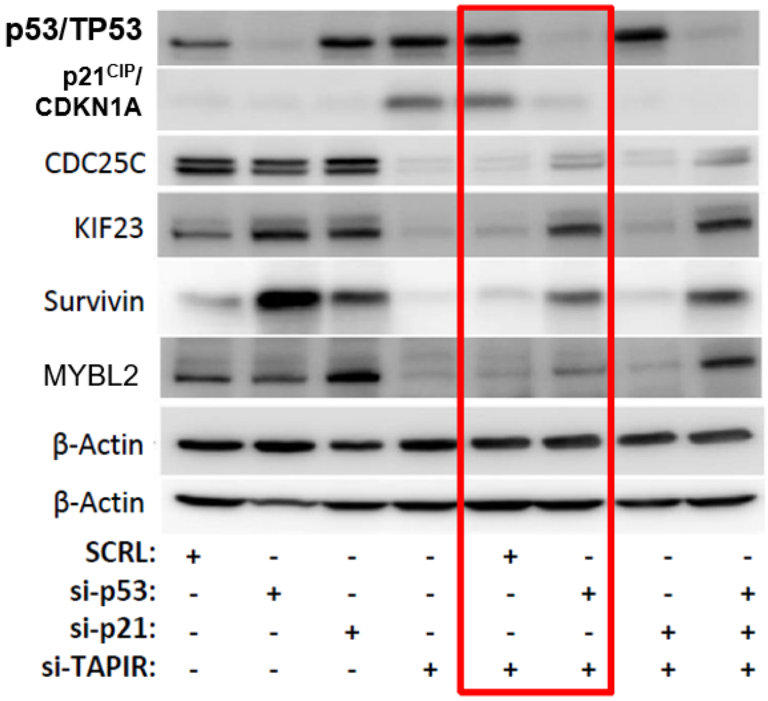
p53/TP53
p21CIP/
CDKN1A
CDC25C
KIF23
Survivin
MYBL2
β-Actin
β-Actin
SCRL: + - - - + - - -
si-p53: - + - - - + - +
si-p21: - - + - - - + +
si-TAPIR: - - - + + + + +

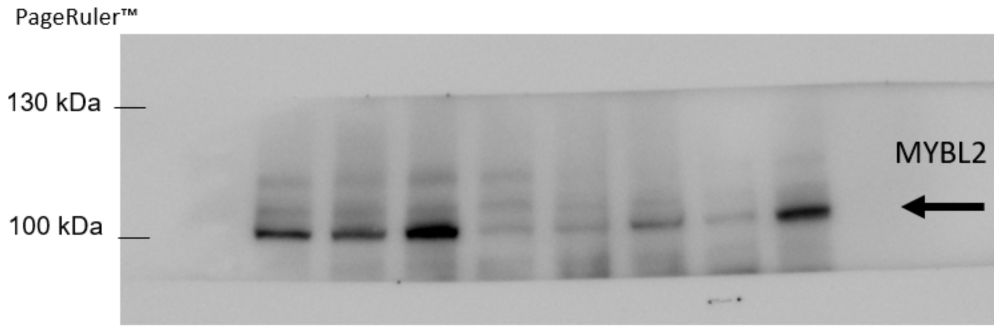
PageRuler™
130 kDa
100 kDa
MYBL2

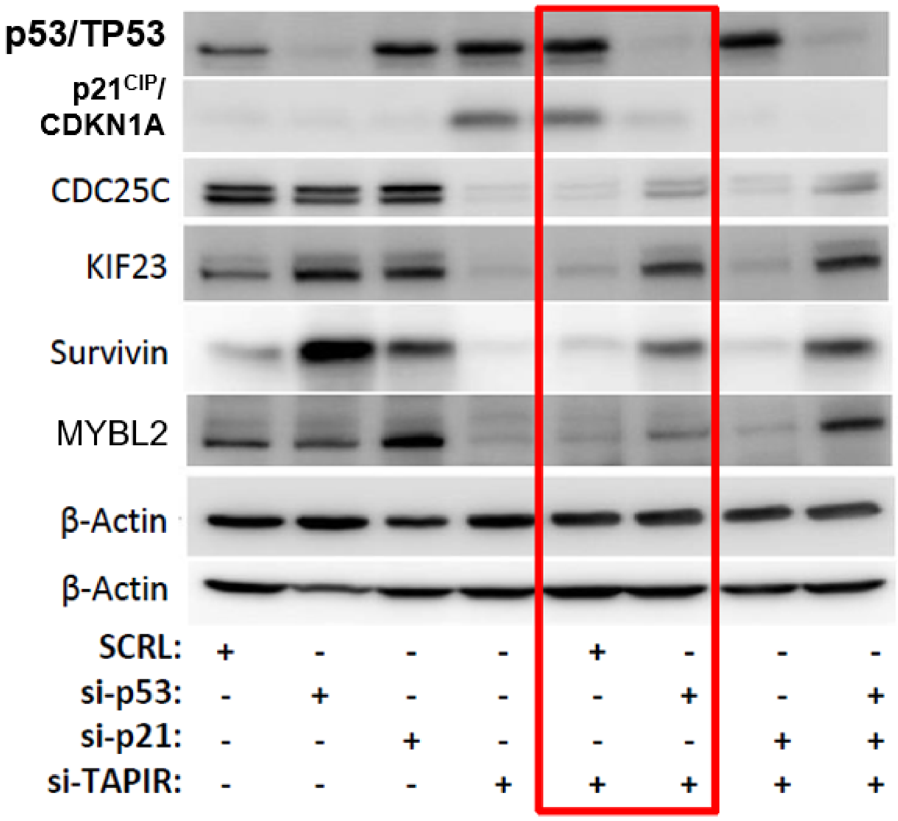
p53/TP53
p21CIP/
CDKN1A
CDC25C
KIF23
Survivin
MYBL2
β-Actin
β-Actin
SCRL: + - - - + - - -
si-p53: - + - - - + - +
si-p21: - - + - - - + +
si-TAPIR: - - - + + + + +

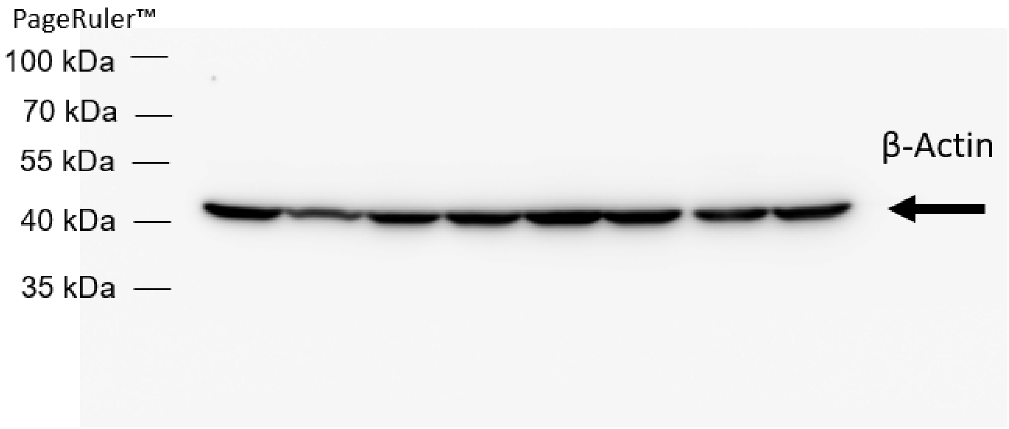
PageRuler™
100 kDa
70 kDa
55 kDa
40 kDa
35 kDa
β-Actin

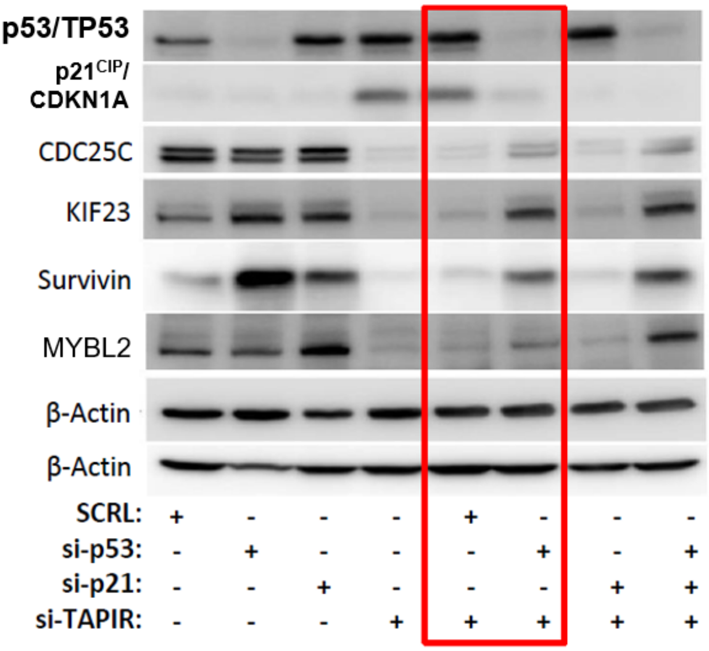
p53/TP53
p21CIP/ CDKN1A
CDC25C
KIF23
Survivin
MYBL2
β-Actin
β-Actin
SCRL: + - - - + - - -
si-p53: - + - - - + - +
si-p21: - - + - - - + +
si-TAPIR: - - - + + + + +

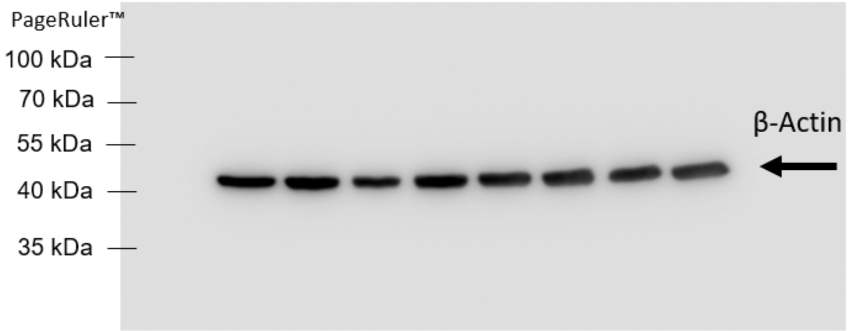
PageRuler™
100 kDa
70 kDa
55 kDa
40 kDa
35 kDa
β-Actin

Table S1. Coding potential prediction of TAPIR-1 and -2 represent non-coding used Coding-Potential Assessment Tool (CPAT).

Name	Sequence ID	RNA size	ORF size	Ficket Score	Hexamer Score	Coding Probability*	Coding Label
TAPIR-1	ENST00000438247.1	786	129	0.5615	-0.1234	0.003	No
TAPIR-2	ENST00000366424.2	1641	339	0.8079	-0.0895	0.070	No

* Based on ORF size, ORF coverage, Fickett TESTCODE statistic, and hexamer usage bias a coding probability below cutoff 0.364 denotes none coding transcripts.

Table S2. Biological Process; GO Term Enrichment analyses of siRNA knockdown experiments in LNCaP cells. Shown are the results (Top 10 table) of si-A1 (*TAPIR-1*) - vs SCBT siRNA treatment 24h post transfection. Graph: Graph: Biological Process; GO Term Enrichment analyses of siRNA knockdown experiments in LNCaP cells. Shown are the results (Top 10 table) of si-A1 (*TAPIR-1*) - vs SCBT siRNA treatment 24h post transfection.

GO.ID	Term	Annotated	Significant	Expected	classicFisher	Adj. p-value
GO:0051276	chromosome organization	1043	123	20.54	1.36e-65	2.08e-61
GO:0007049	cell cycle	1584	135	31.19	2.44e-55	1.87e-51
GO:0022402	cell cycle process	1151	114	22.67	5.96e-52	3.04e-48
GO:1903047	mitotic cell cycle process	718	92	14.14	2.05e-50	7.85e-47
GO:0000278	mitotic cell cycle	869	98	17.11	7.21e-49	2.21e-45
GO:0071103	DNA conformation change	230	56	4.53	1.11e-45	2.84e-42
GO:0006323	DNA packaging	163	48	3.21	1.84e-43	4.03e-40
GO:0007059	chromosome segregation	299	58	5.89	2.58e-41	4.94e-38
GO:0006334	nucleosome assembly	110	40	2.17	1.78e-40	3.02e-37
GO:0031497	chromatin assembly	124	41	2.44	1.71e-39	2.62e-36

Table S3. Molecular Function; GO Term Enrichment analyses of siRNA knockdown experiments in LNCaP cells. Shown are the results (Top 10 table) of si-A1 (TAPIR-1) - vs SCBT siRNA treatment 24h post transfection. Molecular Function; GO Term Enrichment analyses of siRNA knockdown experiments in LNCaP cells. Shown are the results (Top 10 table) of si-A1 (TAPIR-1) - vs SCBT siRNA treatment 24h post transfection.

GO.ID	Term	Annotated	Significant	Expected	classicFisher	Adj. p-value
GO:0046982	protein heterodimerization activity	426	41	8.43	2.92e-17	1.29e-13
GO:0003677	DNA binding	2189	95	43.33	1.13e-14	2.50e-11
GO:0003682	chromatin binding	475	36	9.40	4.11e-12	6.07e-09
GO:0005515	protein binding	9872	244	195.41	2.76e-11	3.05e-08
GO:0046983	protein dimerization activity	1125	52	22.27	6.36e-09	5.64e-06
GO:0031492	nucleosomal DNA binding	33	9	0.65	1.05e-08	7.73e-06
GO:0140097	catalytic activity, acting on DNA	162	17	3.21	2.24e-08	1.23e-05
GO:0042393	histone binding	163	17	3.23	2.45e-08	1.23e-05
GO:1901363	heterocyclic compound binding	5002	144	99.01	2.56e-08	1.23e-05
GO:0035173	histone kinase activity	18	7	0.36	2.92e-08	1.23e-05

Table S4. Cellular Compartment; GO Term Enrichment analyses of siRNA knockdown experiments in LNCaP cells. Shown are the results (Top 10 table) of si-A1 (TAPIR-1) - vs SCBT siRNA treatment 24h post transfection. Cellular Compartment; GO Term Enrichment analyses of siRNA knockdown experiments in LNCaP cells. Shown are the results (Top 10 table) of si-A1 (TAPIR-1) - vs SCBT siRNA treatment 24h post transfection.

GO.ID	Term	Annotated	Significant	Expected	classicFisher	Adj. p-value
GO:0005694	Chromosome	864	124	16.44	5.25e-78	9.98e-75
GO:0044427	chromosomal part	756	115	14.39	1.71e-74	1.62e-71
GO:0044815	DNA packaging complex	85	42	1.62	4.29e-50	2.72e-47
GO:0098687	chromosomal region	290	63	5.52	5.16e-49	2.45e-46
GO:0043228	non-membrane-bounded organelle	3480	179	66.23	2.04e-46	6.48e-44
GO:0043232	intracellular non-membrane-bounded organelle	3480	179	66.23	2.04e-46	6.48e-44
GO:0000228	nuclear chromosome	500	74	9.52	2.60e-45	7.05e-43
GO:0000786	Nucleosome	79	37	1.50	4.88e-43	1.16e-40
GO:0044454	nuclear chromosome part	468	68	8.91	5.92e-41	1.25e-38
GO:0031981	nuclear lumen	3463	168	65.91	8.45e-39	1.61e-36

© 2020 by the authors. Submitted for possible open access publication under the terms and conditions of the Creative Commons Attribution (CC BY) license (http://creativecommons.org/licenses/by/4.0/).
